
JOURNAL INFORMATION
==============================
NLM Title Abbreviation: ScientificWorldJournal
Journal ID: ScientificWorldJournal
NLM Journal ID: 101131163
Journal ID: TSWJ

The Scientific World Journal

ISSN: 2356-6140
EISSN: 1537-744X
Publisher: Wiley

ARTICLE INFORMATION
==============================
PMCID: PMC5956403
PMID: 29861674
DOI: 10.1100/tsw.2004.71
Article ID: 561906
Article version: 1
Subjects: Research Article

Management of Male Infertility

Moreira Sergio G. Jr.
Lipshultz Larry I. *
Division of Male Reproductive Medicine and Surgery, Scott Department of Urology, Baylor College of Medicine, Houston, Texas, USA
*Larry I. Lipshultz: larryl@bcm.tmc.eduAcademic Editor: Anthony Atala

Publication date: 2004
Electronic publication date: 2004 Aug 4
Volume: 4
First page: 214
Last page: 248
Copyright: Copyright © 2004 Jr., Sergio G. Moreira and Larry I. Lipshultz.
Copyright year: 2004
License: This is an open access article distributed under the Creative Commons Attribution License, which permits unrestricted use, distribution, and reproduction in any medium, provided the original work is properly cited.
License URL: https://creativecommons.org/licenses/by/3.0/

==============================
Original Articles
*TheScientificWorldJOURNAL* (2004) 4 (S1), 214–248
ISSN 1537-744X; DOI 10.1100/tsw.2004.71

### Management of Male Infertility

Sergio G. Moreira, Jr., M.D.[1] and Larry I. Lipshultz, M.D.[2,*]
*1Fellow in Male Reproductive Medicine and Surgery; 2Professor of Urology, Chief, Division of Male Reproductive Medicine and Surgery, Scott Department of Urology, Baylor College of Medicine, Houston, Texas*

**E-mail**: smoreira@hsc.usf.edu; larryl@bcm.tmc.edu

*Previously published in the Digital Urology Journal*

---

### GENERAL CONSIDERATIONS

While 85% of couples are able to conceive after one year of protected intercourse, approximately 15% of couples are unable to initiate a pregnancy without some form of assistance or therapy. These patients are said to be "primarily infertile." In approximately one-third of these couples, a male factor appears to be singularly responsible, and in an additional 20% both a male and a female factor can be identified. Therefore, a male factor is at least partly responsible for difficulties in conception in roughly 50% of these couples.

It has been shown that the longer a couple remains subfertile, the worse their chance for an effective cure. In addition, many couples experience significant apprehension and anxiety after only a few months of failure to conceive. For these reasons, unduly prolonged unprotected intercourse should not be advocated before workup of the male is instituted. Although it has often been recommended that clinical evaluation be delayed until 12 months of unprotected intercourse has passed, we believe that the initial screening of the male should be considered whenever the patient presents with the chief complaint of infertility. This initial evaluation, however, should be rapid, noninvasive, and cost-effective. The most important part of the management of male infertility is the correct diagnosis. The use of standard techniques for evaluating medical problems in general, such as complete history, physical examination, and laboratory tests is essential for this purpose.

### INITIAL EVALUATION

### History

A detailed history should address the duration of the couple's infertility, and also previous pregnancies with the present or previous partners. In addition, previous difficulty in achieving conception and any previous evaluation and treatment should be documented (Table 1).

#### *Sexual Habits*

One of the most common problems encountered in this patient population is either too-frequent or too-infrequent intercourse. Often, neither the husband nor the wife understands her menstrual cycle. They do

Correspondence to: Larry I. Lipshultz, M.D., 6560 Fannin, Suite 2100, Houston, Texas 77030; Phone: (713) 798-6163/Fax: (713) 798-6007

**TABLE 1**
**Infertility History**

| History of Infertility | Medical History | Gonadotoxins |
|---|---|---|
| Duration<br><br>Prior pregnancies<br><br>Present partner<br><br>Another partner<br><br>Previous treatments<br><br>Evaluation and treatment of wife | Systemic illness (i.e., diabetes mellitus,<br><br>multiple sclerosis<br><br>Previous/current therapy | Chemicals (pesticides)<br><br>Drugs (chemotherapeutic, cimetidine,<br><br>sulfasalazine, nitrofurantoin, alchohol<br><br>marijuana, androgenic steroids)<br><br>Thermal exposure<br><br>Radiation<br><br>Smoking |
| **Sexual History** | **Surgical History** | **Family History** |
| Potency<br><br>Lubricants<br><br>Timing of intercourse<br><br>Frequency of intercourse<br><br>Frequency of masturbation | Orchiectomy (testis cancer, torsion)<br><br>Retroperitoneal injury<br><br>Pelvic injury<br><br>Pelvic, inguinal, or scrotal surgery<br><br>Herniorrhaphy<br><br>Y-V plasty, transurethral resection of the<br><br>prostate | Cystic fibrosis<br><br>Androgen receptor deficiency<br><br>Infertile first-degree relatives |
| **Childhood & Development** | **Infections** | **Review of Systems** |
| GU congenital anomalies<br><br>Undescended testes, orchiopexy<br><br>Herniorrhaphy<br><br>Y-V plasty of bladder<br><br>Testicular torsion<br><br>Testicular trauma<br><br>Onset of puberty | Viral, febrile<br><br>Mumps orchitis<br><br>Venereal<br><br>Tuberculosis, smallpox (rare) | Respiratory infections<br><br>Anosmia<br><br>Galactorrhea<br><br>Impaired visual fields |

Modified from Sigman, Lipshultz, L.I., and Howards, S.S.: Evaluation of the subfertile male. In: *Infertility in the Male*, 3rd edition. Edited by L.I. Lipshultz and S.S. Howards. St.Louis: Mosby-Year Book, 1997, p.174.

not realize that the optimal time for intercourse is midcycle and that the most effective frequency of intercourse is every 48 hours. This is based on the fact that sperm survival in normal cervical mucus and within the cervical crypts is approximately 2 days. Thus, this frequency will assure viable spermatozoa concurrently in the 24-hour period during which the egg will be within the fallopian tube and capable of being fertilized.

It is also important to discuss coital techniques with the husband, e.g., the use of lubricants or the frequency of masturbation that can deplete the sperm "reserve." Many lubricants have been tested for *in vitro* effects on sperm motility.[1] Commonly used substances, such as K-Y Jelly, Lubifax, Surgilube, Keri Lotion, petroleum jelly, and saliva result in a deterioration of motility. Others, such as raw egg white, vegetable oil, and the Replens douche, do not impair *in vitro* motility. Astroglide, a water-soluble, inert vaginal lubricant, contains no petroleum ingredients or detergents that may be toxic to sperm; however, with increasing concentration, there is impairment of sperm motility equivalent to that found with K-Y jelly.

### Childhood Illnesses

A history of specific childhood illnesses and disorders may be an important finding in the evaluation of the infertile male. For example, it has been shown that in the male born with a unilaterally undescended testis, regardless of the time of orchiopexy, overall semen quality is considerably less than that found in normal men. Approximately 30% of men with unilateral cryptorchidism and 50% with bilateral cryptorchidism have sperm densities below 12-20 million/mL.[2] Despite this impairment in semen parameters, the majority of men with a history of one undescended testis are able to initiate a pregnancy without difficulty.

Testicular trauma or torsion of the testes should be noted, since both can result in atrophic testes. Approximately 30% of men with history of testicular torsion will have abnormal results on semen analysis.[3]

A history of postpubertal mumps orchitis is also important. Mumps does not appear to affect the testes when experienced prepubertally. However after the age of 11 or 12, unilateral mumps orchitis is seen in 30% of males affected and bilateral orchitis in approximately 10%.[4] Furthermore, the testicular damage can be quite severe and should be readily appreciated on physical examination, since the involved gonads will be markedly atrophic.

Patients who have had operative correction (Y-V plasty) of their bladder neck during childhood often suffer from retrograde ejaculation due to ablation of the internal sphincter. Bladder neck reconstruction at the time of ureteral reimplantation surgery was common in the early 1960s; this patient population has now entered an age group when pregnancy will most likely be attempted. Retrograde ejaculation should be suspected in the man who gives a history of bladder surgery and whose ejaculate volume is less than 1 cc, severely oligospermic, and abnormally alkaline. The correct diagnosis can be made by finding large numbers of sperm in the postejaculate urine. Children born with congenital anomalies involving the male reproductive system, such as bladder exstrophy/epispadias, can also exhibit abnormalities of ejaculation because of difficulties with both intromission and ejaculation. Spermatogenesis is usually normal; however, the ejaculatory ducts may be obstructed, or retrograde ejaculation may occur. Also, a history of herniorrhaphy suggests the possibility of iatrogenic vasal injury.

### Exogenous Agents That Interfere With Spermatogenesis

The history should also include a detailed inquiry into exposure to environmental toxins and medications that may interfere with spermatogenesis, either directly or through alterations in the endocrine system. For agents such as heat, ionizing radiation, heavy metals, and some organic solvents, there are many studies that support these associations. Recent publications have also reported the effect of specific pesticides (i.e., dibromochloropropane) on gonadal function.[5] Furthermore, reversibility has been substantiated when the oligospermic patient has been removed from this toxic environment.[6] However, once azoospermia has occurred, return to a normal pre-exposure state is highly unlikely.

Medications, such as sulfasalazine and cimetidine, or ingestants, such as caffeine, nicotine, alcohol, or marijuana, have also been implicated as gonadotoxic agents. Withdrawal from these substances should enable return of normal spermatogenesis if they are acting adversely. Also, calcium ion channel blockers may interfere with sperm membrane function and fertilization ability.[7]

The use of androgenic steroids by athletes is a potentially significant cause of infertility in both adults and adolescents, and the problem is becoming more commonplace.[8] The incidence of steroid abuse has been reported to be as high as 30%-75% among professional athletes or body builders. Androgenic steroids exert their deleterious effect by depressing gonadotropin secretion and interfering with normal spermatogenesis. Consequently, if a person is taking any of these medications at the time of initial interview, the medication should be stopped and the patient's semen reevaluated at a later date.

Elevated temperatures, as in the routine use of saunas and hot tubs, may interfere with spermatogenesis.[9]

### Surgical History

***Retroperitoneal Lymph Node Dissection:*** Approximately 75% of all testicular cancer patients will retain the potential for fertility.[10] Retroperitoneal lymph node dissection can involve excision of portions of the sympathetic chain necessary for ejaculation. Some patients will retain seminal emission, but many will have retrograde ejaculation or lose the ability to emit semen altogether.

***Prostatectomy:*** Patients who have had transurethral or open prostatectomy also have a high incidence of retrograde ejaculation. This incidence is reported to range from 40%-90%.

### Physical Examination **(Table 2)**

Physical examination of the infertile man should include a generalized and complete evaluation. Any factor that affects overall health can theoretically be responsible for abnormalities in sperm production. For that reason, the physical examination should be thorough, with emphasis placed on the genitalia.

### Body Habitus

If the patient appears to be inadequately virilized (androgen-deficient), as evidenced by decreased body hair, gynecomastia, eunuchoid proportions, etc., the diagnosis of delayed maturation due to an endocrine abnormality should be considered and evaluated.

### Phallus

Penile curvature or angulation should be assessed, as should the location of the urethral meatus, i.e., for presence of hypospadias. Abnormalities can result in improper placement of the ejaculate within the vaginal vault.

**TABLE 2**
**Physical Examination**

| | |
|---|---|
| **Body Habitus** | Decreased body hair<br>Gynecomastia<br>Eunuchoid proportions |
| **Phallus** | Peyronie's disease<br>Congenital curvature<br>Hypospadias |
| **Scrotum** | Testicular volume<br>Epididymal induration<br>Presence/absence of vas deferens<br>Varicocele |
| **Digital Rectal Examination** | Prostate size<br>Prostatic/seminal vesicular mass/induration/cysts |

### *Scrotum*

The scrotal contents should also be carefully palpated with the patient standing. Testicular size and consistency must be noted and the volume of the testis estimated either with an orchidometer or by measuring the long and wide diameter of the testes to the nearest millimeter. It has been shown that a decrease in testicular size is often associated with impaired spermatogenesis. Standard values of testicular size have been recorded for the normal population.[11] These data document that in the normospermic male, the length of the testis should be greater than 4 cm and the volume greater than 20 mL.

Examination of the peritesticular area is also essential. Epididymal induration, irregularity, and cystic changes should be noted, as should the presence absence of the vas deferens and any nodularity along its course. Certainly, engorgement of the pampiniform plexus should be identified, since a varicocele can cause abnormalities of gonadal function.[12] Ideally, the patient should be examined in a warm room after standing for several minutes. Palpation for asymmetry of the spermatic cords, followed by a Valsalva maneuver with re-palpation of the spermatic cords, should be routinely performed. An "impulse" can often be felt with the increase in intra-abdominal pressure.

### *Digital Rectal Examination (DRE)*

DRE is necessary to assess prostatic size, as well as to rule out prostatic and/or seminal vesicular induration, masses, or cysts.

### LABORATORY ASSESSMENT

### Routine Laboratory Tests

### *Semen Analysis*

Once an appropriate history and physical examination have been completed, appropriate laboratory testing should be performed. Certainly, the central component of the laboratory evaluation is the semen analysis. Nevertheless, it must be emphasized that the semen analysis is not a test for fertility. Fertility determination is a couple-related phenomenon that requires the initiation of a pregnancy. A female factor evaluation should be completed concurrently with that of the male to achieve optimal results.

All specimens must be collected with a consistent abstinence period of 2-3 days and brought to the laboratory for evaluation of sperm motility and forward progression within 2 hours of collection. The specimen container should be clean, but not necessarily sterile. Some plastics contain residual spermatocidal chemicals and should be checked to rule out spermatotoxic contaminating material. Collection of the semen can be by masturbation, coitus interruptus, or with a special condom devoid of spermatocidal agents.

It is important for the clinician to understand the difference between "normal" (average) and "adequate" (potentially fertile) semen quality. If one could measure semen quality in a non-biased population of patients, it would be possible to calculate a mean or average sperm density. However, these data have been difficult to obtain. A compilation of previous studies shows that the authors are not in agreement. Most of these patients are from vasectomy clinics, i.e., a setting where the critical period of sexual abstinence could not be controlled and where the motivation for obtaining a complete ejaculate was not high. In addition, the mean age of this population is higher than that of men presenting for routine fertility evaluation.

Clinical studies of infertile patients have also established limits of adequacy, below which initiation of a pregnancy becomes statistically increasingly difficult (Table 3). World Health Organization (WHO) standards are slightly, though not significantly, different (Table 4). Nevertheless, men with high-quality spermatozoa may be fertile with very low sperm densities.

**TABLE 3**
**Semen Analysis: Minimal Standards of Adequacy**

| | |
|---|---|
| ***On at least two occasions:*** | |
| Ejaculate volume:<br>Sperm density:<br>Motility:<br>Forward progression:<br>Morphology: | 1.5-5.0 mL<br>>20 million/mL<br>>60%<br>>2 (scale 0-4)<br>>60% normal |
| ***And:*** | |
| No significant sperm agglutination<br>No significant pyospermia<br>No hyperviscosity | |

Modified from Sigman, Lipshultz, L.I., and Howards, S.S.: Evaluation of the subfertile male. In: *Infertility in the Male*, 3rd edition. Edited by L.I. Lipshultz and S.S. Howards. St.Louis: Mosby-Year Book, 1997, p.174.

### *Basic Laboratory Tests*

Laboratory investigation of testicular function begins with basic screening tests. The tests to be performed will depend on the clinical history and physical examination. The hormones most frequently measured are testosterone (T), luteinizing hormone (LH), and follicle-stimulating hormone (FSH).

Serum T reflects Leydig cell function and provides an easy available indicator of intratesticular testosterone. Measurement of circulating LH and FSH levels allows the clinician to determine if a patient's endocrine dysfunction is the result of primary testicular failure or hypothalamic and/or pituitary deficiency.

**TABLE 4**
**World Health Organization (WHO) Criteria For Normal Semen Values**

| | |
|---|---|
| **Volume** | >/= 2.0 cc or greater |
| **pH** | 7.2-7.8 |
| **Sperm concentration** | >/= 20 million/cc |
| **Total sperm count** | >/= 40 million |
| **Motility** | >/= 50% with normal morphology |
| **Morphology** | > 30% normal forms |

Modified from Gilbert, B.R., *et al*.: Semen analysis in the evaluation of male factor subfertility. AUA Update Series, Lesson 32, VolumeXI, 1992.

Other tests are indicated in certain circumstances. Serum prolactin should be measured in patients with signs and symptoms suggestive of a pituitary tumor, in any patient with a low serum testosterone level without an associated increase in serum LH, and in patients taking psychotropic drugs or centrally acting antihypertensives.

An assessment of other pituitary hormones (adrenocorticotropic hormone [ACTH], thyroid-stimulating hormone [TSH], and growth hormone [GH]) is recommended in all patients with hypogonadotropic hypogonadism (LH and FSH deficiency).

### Additional Laboratory Tests

Between 10% and 20% of infertile couples evaluated will be found to have "unexplained" infertility. In the female, this percentage is rapidly decreasing as more sophisticated techniques have been developed to accurately identify the efficacy of evaluation. In the male, we have only recently come to realize that sperm number and motility, as determined by routine semen analysis, do not define function or true sperm quality. Consequently, additional tests have been developed to identify other abnormalities of semen parameters. These include leukocyte and antisperm antibody identification, as well as tests of sperm function.

### *Quantitation of Leukocytes in Semen*

Recently, monoclonal antibody technology has been applied to the identification of leukocytes in semen. Some patients presenting for infertility demonstrate numerous round cells in their semen. With a standard semen analysis, it is very difficult to differentiate between immature germ cells and leukocytes. Since pyospermia may indicate an infection, it is useful to be able to determine the cell type. Recent work by Anderson has shown that infertile men have higher white blood cell counts in their ejaculates than normal men.[13]

### *Antisperm Antibody Testing*

The presence of antisperm antibodies (ASA) in the semen often correlates with lower pregnancy rates. Such a condition may be suggested by several risk factors, including prior genital infections, testicular trauma or biopsy, heat-induced testicular damage, or genital tract obstruction (Table 5). ASA may also be suspected on semen analysis with clumping/agglutination, diminished motility, a poor postcoital test, or the presence of the "shaking" phenomenon on sperm-cervical cross-mucus testing.

**TABLE 5**
**Risk Factors in the Development of Antisperm Antibodies (ASA)**

| | |
|---|---|
| **Obstruction:**<br>Vasectomy<br>Vasectomy reversal<br>Vasal or seminal vesicle agenesis<br>Testicular<br>**Infection:**<br>Orchitis<br>Venereal disease<br>Prostatitis<br>**Thermal:**<br>Varicocele<br>Cryptorchidism | **Physical Injury:**<br>Trauma<br>Torsion<br>Biopsy<br>Coitus<br>**Genetic:**<br>Human lymphocyte antigens |

Modified from Turek, P.J.: Immunopathology and infertility. In: *Infertility in the Male,* 3rd Edition. Edited by L.I. Lipshultz and S.S. Howards. St. Louis: Mosby-Year Book, 1997, p.313.

Although several different methods to detect these antibodies have been described, the most accurate assay currently appears to be the immunobead test. This assay utilizes polyacrylamide beads to which rabbit antihuman antibodies have been linked. It can accurately detect IgA or IgG antibody binding to the head, midpiece, or tail of motile sperm. A finding of more than 20%-50% of sperm demonstrating immunobead binding is felt to be clinically significant. Tests that measure antisperm antibodies in the serum or seminal plasma are less useful than sperm-bound antibody assays, as it is the sperm surface antibodies that most likely product the functional deficits associated with immunologic infertility.

### Advanced Sperm Fertility Tests

There is no single sperm assay that is a global indicator of male infertility. Therefore, to assess properly the ability of the spermatozoon to fertilize, one must optimally use a combination of tests and be aware there is no widely accepted standard advanced sperm function test that is currently in use. Also, one must be clear regarding the purpose for semen measurements and sperm function tests.

### *(a) Strict Morphology*

Traditionally, the semen analysis included the percentage of spermatozoa with normal morphology, and if this percentage was below the normal threshold, these patients were considered to be teratozoospermic. In 1986, Kruger et al. introduced "strict" criteria with a clinically significant threshold of 14% normal forms.[14] These criteria were based on measurements taken from spermatozoa that successfully migrated to the cervix. Normal sperm morphology, as assessed by these strict criteria, is an excellent predictor of *in vitro* fertilization (IVF) rates. The IVF rates were substantially reduced in individuals with less than 14% morphologically normal spermatozoa and reduced even more when this percentage was less than 4%.

To assess sperm morphology, spermatozoa are fixed with the Papanicolaou stain, and at least 200 spermatozoa per slide are analyzed. The criteria for the normal spermatozoon include a smooth, oval sperm head measuring 3-5 micrometers in length and 2-3 micrometers in width. There should be no defects of the neck, midpiece, or tail, and a well-defined acrosome should comprise 40%-70% of the sperm head (Fig. 1). There should be no cytoplasmic droplets larger than half the size of the sperm head.

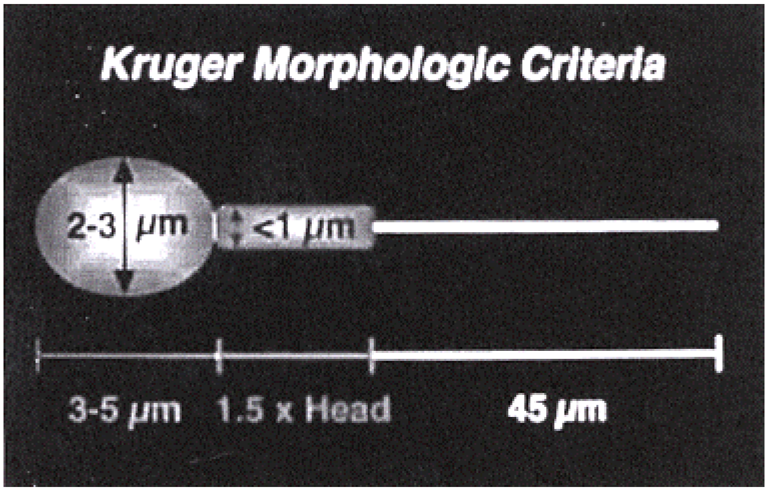

**FIGURE 1.** Criteria for Kruger strict morphology.

All borderline forms are classified as abnormal. Abnormal head forms include tapered, pyriform, duplicated, macro, micro, and amorphous. Other modified versions of strict criteria have also been described. Notably, WHO has recently issued stricter criteria and lowered the reference value of normal sperm forms of sperm to an empirically set minimum of 30%.

Advocates feel that this simple, rapid, and inexpensive test can be most useful as a predictor of IVF rates and in optimizing IVF rates by increasing the concentration of inseminated spermatozoa for teratozoospermic patients. Currently, many investigators have adapted strict morphology as a routine laboratory sperm test.

### (b) Computer-Assisted Semen Analysis (CASA)

CASA was introduced in the 1980s to provide an automated, objective, and standardized evaluation of sperm concentration and movement. The variables measured by most CASA systems are sperm density, percent motility, straight-line velocity, curvilinear velocity, linearity, average path velocity, amplitude of lateral head displacement, flagellar beat frequency, and hyperactivation This technology is based on digitalized sperm images that are visualized by a video camera and analyzed by a computer.

Disadvantages of CASA include standardization of specimen preparation, cost, technician expertise, and an understanding of the limitations of computer-based analysis. In addition, CASA can be highly inaccurate when measuring spermatozoa at very high or very low concentrations. Routine use of CASA in the andrology laboratory is controversial in part because of a lack of understanding of the specifications and limits of this equipment and also because in the majority of cases CASA may offer little clinical advantage over routine semen analysis.

### (c) Hypo-Osmotic Swelling Test (HOS)

In 1984, Jeyendran et al. reported that under hypo-osmotic conditions (150 mOsm/L), a normal spermatozoon will absorb fluid resulting in bulging of the plasma membrane and curling of its tail.[15] This test is based upon the principle that a living spermatozoon can maintain an osmotic gradient whereas a dead cell cannot.

This curling is readily detected by using phase-contrast microscopy (Fig. 2). This simple test measures the physical and functional integrity of the plasma membrane and therefore viability. In an

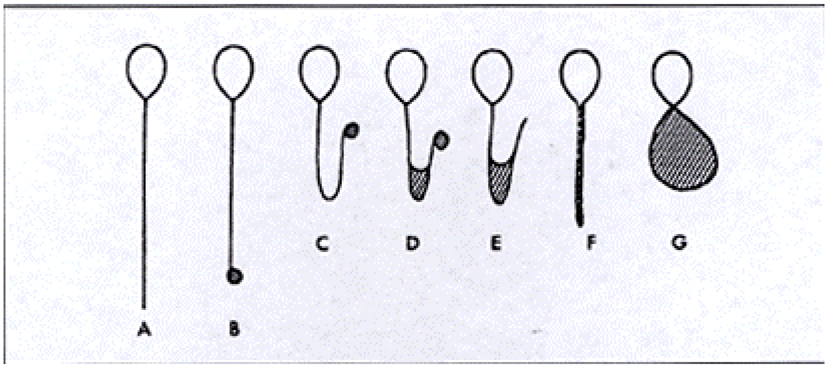

**FIGURE 2**. Hypo-osmotic swelling of spermatozoa. Hatched areas indicate swelling. A, no tail swelling and an abnormal response. B-G, various types of tail swelling and a normal response. (From Jeyendran RS, et al: J. Reprod. Fertil., 70:219, 1984.)

abnormal sample, less than 50% of spermatozoa swell; in a normal one, more than 60% of spermatozoa react. The investigators believe that when performed properly, this test provides functional information independent of other fertility tests and is particularly useful when no swelling is seen, correlating in this instance with very poor IVF results. This assay can differentiate immotile but viable spermatozoa from necrospermia. The HOS test is currently used most often in selecting live testicular sperm intracytoplasmic sperm injection (ICSI), where there is little or no motility.

### (d) Viability Stain Assays

Viability stains are also used to determine if spermatozoa are alive and if the plasma membrane is intact. These tests are based on the principle that live cells can exclude dye whereas damaged dead cells cannot. The stains used are eosin Y and trypan blue. The vitality results of the stain assay and of the HOS test correlate very closely, since both evaluate the integrity of the plasma membrane. Similar to the HOS test, these assays add little to predicting prognosis of IVF results and determining a diagnosis, except in cases with very low or absent motility (by differentiating it from necrospermia). Unfortunately, once sperm are stained, they are no longer viable and cannot be used for ICSI.

### (e) Cervical Mucus/Sperm Interaction Assays

The spermatozoa must travel through the cervical mucus to reach the uterus. Failure of passage through the cervical mucus is the primary cause of infertility for 10% of couples consulting for this condition. The quality of the cervical mucus varies during the menstrual cycle.

The postcoital test (PCT), first performed by Sims more than 125 years ago, has traditionally been a common way to determine cervical mucus/sperm interaction. This test evaluates sperm concentration and motility in an aspirate of cervical mucus at midcycle shortly after the couple has intercourse. Results of a normal PCT would show the presence of 20 or more spermatozoa per high-power field. An abnormal PCT results most commonly is secondary to inappropriate timing of coitus. Other causes include ASA, anovulation, an abnormal hormonal milieu, female or male genital tract infections, poor semen quality, and male sexual dysfunction.

Because this test relies heavily on factors that are beyond the control of the clinic, the usefulness of the PCT in infertility investigation has been questioned. Whereas the presence of motile spermatozoa indicates that spermatozoa can survive in the cervical mucus, failure to find motile spermatozoa is more difficult to interpret.

Tests that investigate the *in vitro* interaction between spermatozoa found in semen and sperm-free midcycle mucus are also clinically useful. *In vitro* tests, such as the capillary test, were introduced in an attempt to standardize the sperm-mucus penetration capacity. The crossed mucus-hostility assay, which uses donor spermatozoa and mucus as controls, is utilized to determine if it is the male or female partner who is responsible for poor sperm-cervical mucus interaction. Recently, a commercial assay (Penetrak test) has been developed using bovine cervical mucus that is similar to human cervical mucus both biochemically and physiologically. However, this assay does not assess the female component of the cervical factor. The Tru-Trax Assay (Humagen) combines both approaches, placing human and bovine cervical mucus in adjacent wells. Niederberger *et al*. have shown reliability and reproducibility in both assays.[16] Some investigators have reported a poor correlation of cervical mucus penetration assays with IVF and pregnancy rates, while others refute this. There is also disagreement of whether these assays correlate with one another, with motility and other semen variables. It is likely that the cervical mucus penetration test measures a sperm function that is independent of other sperm functions measured.

### (f) Sperm Capacitation Assays, Mannose-Ligand Receptor Assays, and Acrosome Reaction Assays

Currently, these tests are more commonly used for research purposes.

### (g) Sperm Penetration Assay (SPA)

The SPA was developed to measure the functional properties of sperm and was initially developed following the observation that, upon the removal of the zona pellucida of hamster ova, the species specificity of fertilization and the block to polyspermy are lost. In particular, heterologous penetrations between hamster ova and sperm from a variety of species, including humans, has been observed. Ideally, human ova should be used for this assay, but they are not widely available, and there are ethical problems associated with their use. Therefore, hamster ova have provided a useful model for the measurement of human sperm function.

For fertilization to occur *in vivo*, the sperm must first be capacitated and have undergone the acrosome reaction. The physiology of sperm capacitation is not clearly defined. In particular, it is not known whether capacitated sperm that have gained the ability to penetrate human ova have undergone the acrosome reaction, or whether this occurs as a local event at the time of gamete fusion.

The use of SPA as a measure of potential fertility is based on the theory that fertile sperm samples will either penetrate most hamster ova or result in a significant amount of polyspermy of the penetrated ova. Infertile sperm samples are expected to penetrate a lower percentage of ova or result in a lesser degree of polyspermy.

Consideration should be given to obtaining the SPA in couples with unexplained infertility or in couples in whom the decision is being made to proceed with intrauterine insemination (IUI) or IVF, since lower SPA results have been predictive of poor success with IVF and lower pregnancy rates in couples attempting conception through intercourse.[17]

### (h) Reactive Oxygen Species (ROS) Assay

For cells living under aerobic conditions, oxygen represents a paradox: While it is required for survival and normal function, its metabolites can be potentially toxic due to the generation of oxygen-free radicals. Some of these metabolites, called ROS, have been shown to be produced by spermatozoa and to generate toxic effects on sperm function. However, when produced at the right time and amount, these ROS can also initiate and promote normal physiologic reactions such as sperm hyperactivation and capacitation. In human semen, high ROS formation was detected in 40% of semen samples from an unselected population of men consulting an infertility clinic.

### Genetic Evaluation

Genetic abnormalities related to male infertility need to be considered in terms of being (1) causative for male infertility and (2) potentially transmissible to the offspring. Reasons for pursuing a genetic evaluation include (1) establishing a diagnosis, (2) establishing the genetic origin of the abnormality, (3) clarifying the pattern of inheritance, and (4) providing information on natural history, variation, and expression. When examining the offspring, the physician should keep in mind that abnormalities may be transmitted from either parent, or may arise *de novo*, depending on the specific defect. The three most common known genetic factors related to male infertility are cystic fibrosis (CF) gene mutations leading to congenital absence of the vas deferens, Y-chromosome microdeletions leading to spermatogenic impairment, and karyotype abnormalities.

When a man has azoospermia, it is the responsibility of the urologist to determine whether testicular failure or obstruction is present. If the vas deferens is not palpable, unilaterally or bilaterally, then CF gene mutation testing is necessary. The most commonly encountered condition in this category is cystic fibrosis transmembrane conductance regulator (CFTR) gene mutations, which typically manifest in men with azoospermia and vasal or epididymal abnormalities. The most common manifestation is congenital bilateral absence of the vas deferens, which occurs in 1%-2% of men presenting with infertility. The carrier status for this autosomal recessive condition is quite common, being present in 1/25 persons of Northern European descent and with over 550 CFTR gene mutations having been reported. Because of the potentially fatal nature of this autosomal recessive disorder for the offspring, screening should now be considered routine when vasal or epididymal abnormalities are suspected.

Deletions in the Y chromosome (i.e., DAZ, YSRRM, and AZF a, b, c deletions) are now known to result in severe oligospermia and azoospermia. Consequently, a genetic etiology of male factor infertility must be considered in these patients. At our institution, in those patients with a sperm density of less than 10 million/mL, we send peripheral blood for Y chromosome analysis. A positive analysis has implications not only for diagnosis, but in the age of intracytoplasmic sperm injection (ICSI), for potential transmission of subfertility to a male offspring. The role for chromosomal analysis (+/- painting) has yet to be defined for patients undergoing ICSI but should be performed in cases of ICSI failure.

Karyotyping can uncover genetic abnormalities in the infertile male including structural chromosomal disorders such as Klinefelter's (classic 47,XXY), mixed gonadal dysgenesis, chromosomal translocations, and XYY syndromes. Chromosomal abnormalities are not uncommon in the general population, as the incidence has been estimated at 0.5%. Klinefelter's syndrome is relatively common with an incidence of 1 in 500 live male births and is the most common abnormality of sexual differentiation, while the XYY male syndrome occurs in about 1 in 1000 live births.[18] A great majority of these patients are fertile, with infertility being as common as the normal population. Identification of these disorders is important because with the advent of ICSI, men with Klinefelter's syndrome (mosaic and non-mosaic) can have sperm harvested from testis biopsies and initiate a pregnancy with resultant genetically normal embryos[19]. Men with Klinefelter's may also have severe oligozoospermia.

### SURGICAL TREATMENT OF THE INFERTILE MALE

### Diagnostic Studies

### *(a) Transrectal Ultrasound (TRUS)*

TRUS offers a noninvasive means of studying both the prostate and the accessory sex glands. The indications for TRUS remain controversial, but standard criteria include low volume and acidic azoospermic semen specimens. These findings suggest absence of seminal vesicle fluid in the semen consistent with complete ejaculatory duct obstruction. Also, dilated seminal vesicles are suggestive of ejaculatory duct obstruction due to a midline cyst, which may respond to a transurethral resection of the ejaculatory ducts (TURED). Ultrasound guidance may be used during needle aspiration of the seminal vesicles, which may help determine if there is ejaculatory duct obstruction.

### (b) Vasography

Vasography can be performed at the time of testicular biopsy if normal spermatogenesis is demonstrated. A transverse micro-incision in the vas near the junction of the straight and convoluted portions will allow immediate examination of the effluxing fluid for the presence of sperm as well as localization of the level of obstruction. The use of optical magnification (we use the operating microscope) is advised.

Sterile saline may be injected distally in an antegrade direction to assess vasal patency. If resistance is encountered, distal obstruction must be ruled out. Chromotubation or antegrade injection of indigo carmine into the vas deferens is next performed. The urethra is then catheterized to check for blue-stained urine. The absence of blue urine confirms distal vasal obstruction. The site of vasal obstruction may then be documented by passing a #0 Prolene suture in an antegrade direction through the vas and determining the distance from the vasotomy to the level of the obstruction. If the level of obstruction remains unclear, an antegrade vasogram, using sterile Hypaque or Renografin solution, will demonstrate the anatomy of the distal vasal obstruction. Retrograde injection should be avoided as epididymal trauma may result. In an effort to avoid leakage or stricture formation, a microsurgical technique should be used to repair the vasotomy site, i.e., closure with 10-0 and 9-0 monofilament microsutures.

### (c) Testis Biopsy

Since the early reports by Charny and Hotchkiss in the 1940s,[20,21] the importance of testis biopsy in the male infertility evaluation has been recognized. Testis biopsy is important in the azoospermic patient for differentiating obstruction from nonobstructive testicular pathology. In addition, in the patient with severe, unexplained oligospermia, a testis biopsy can provide a tissue diagnosis that will furnish prognostic information and help direct future medical treatment.[22] Bilateral biopsies may be indicated if there is a suggestion of an asymmetrical testicular lesion, such as ductal obstruction with primary testicular failure.[23] Testis biopsy can also be used for "mapping" of the testes for later sperm aspiration for ICSI.[24] Elevated serum gonadotropins should not preclude direct scrotal investigations; surprisingly good sperm production may be present in the setting of very high FSH values. In addition, testicular biopsy for screening of intratubular germ cell neoplasia or carcinoma *in situ* (CIS) is of relevance to the infertile population, since an increased incidence of CIS is found in men with a history of cryptorchid testes.[25]

The technique of open testis biopsy has been well described in many texts. Local anesthesia using a cord block and local infiltration often with mild sedation may be utilized. A general anesthetic is preferable if extensive scrotal exploration with concurrent microsurgical correction is indicated.

A "window" technique provides the simplest approach and should be used if obstruction is not suspected. The anterior scrotal skin is stretched while the testis is lifted, and a 1-2 cm incision is made through the tunica vaginalis. Small hemostats placed on the edges of the tunica vaginalis offer easier closure. If local anesthesia is being utilized, Lidocaine may then be dripped onto the surface of the tunica albuginea. Placement of a small eyelid retractor may improve exposure at this point. After the tunica albuginea is incised for approximately 5 mm with a #11 scalpel blade, gentle pressure on the testis will extrude the seminiferous tubules. With a no-touch technique, the specimen is excised with sharp Iris scissors and promptly placed in Bouin's, Zenker's, or buffered glutaraldehyde solution. Ten percent formalin, used in most types of tissue fixation, will introduce distortion artifacts in the testis biopsy and should not be used. Prior to placing the specimen into the solution, a touch preparation slide can be made for immediate review, i.e., testicular cytology.

Hemostasis is obtained with careful use of electrocautery, and the tunica albuginea is closed with fine, absorbable suture as are the layers of the scrotum. If there is no intention to repair an obstructive ductal lesion, the "window" technique is preferable to exposing the entire testicle, which will encourage the formation of adhesions; this makes future exploration more difficult. If epididymal obstruction is suspected, the testis should be introduced into the operative field through a more generous scrotal incision.

***Testicular Needle Biopsy:*** Several reports have appeared discussing the relative merits of needle versus open testis biopsy. The performance of needle biopsy suggests that the study can be performed as an office procedure, with little pain and low morbidity, and yields adequate information. Techniques have been described using the Vim-Silvermann[26] or Tru-Cut biopsy needle[27] to obtain a core of tissue or using fine-needle aspiration with material smeared on the microscope slide. A high degree of correlation with the findings on routine histologic section has been demonstrated. However, tissue sampling is not really as comprehensive. One potential use of needle biopsy would be to document active spermatogenesis in an azoospermic patient in the office setting, so that subsequent operative exploration with microsurgical capability can be planned.

***Testicular Fine-Needle Aspiration (FNA) Cytology:*** FNA cytology of the testis has also been described extensively and has been described as a minimally traumatic procedure having high correction with histologic studies.[28] Because of the "heterogenous picture" of the spermatogenic process within the testis, a single aspirate or biopsy may not be truly reflective of the entire testis.[29] Testicular FNA has not gained widespread acceptance in the evaluation of the infertile male for numerous reasons. Although cellular detail is excellent, information regarding peritubular fibrosis, the interstitial tissue, and cellular arrangement is lacking.

***Testicular Cytology:*** We have been studying the use of two techniques that allow cytologic analysis of the germinal epithelium using a testis biopsy.[30] These are the "touch imprint" and ìcytospinî preparations. The former has proved the easiest with clear cytologic findings. An open biopsy specimen is touched to or gently smeared across a microscopic slide, which is then rapidly sprayed with cytofixative and stained with routine hematoxylin and eosin (H&E). A cytospin is prepared by immersing the specimen in cold tissue culture solution and agitating gently for 1 minute. The specimen is then removed and placed in an appropriate fixative for routine processing, while the cold solution containing cells that have washed out of the cut tubules is centrifuged on a cytospin processor and stained.

These methods provide a rapid means of examining the cellular contents of the seminiferous tubules. Because mature, intact spermatozoa are clearly identified in this manner, we have found such cytologic preparations useful in distinguishing late maturation arrest from normal biopsies; this is often difficult on frozen or paraffin sections. We have also found cytology to be the most rapid means of confirming intraoperatively that complete spermatogenesis is occurring.

### Procedures to Improve Sperm Production

### *(a) Varicocele Detection*

A varicocele is defined as a dilatation of the pampiniform venous plexus that surrounds the testis. The surgical correction of a varicocele, known as varicocelectomy, is the most commonly performed operation for the treatment of male infertility.

***Physical Examination:*** Careful physical examination is mandatory for the accurate diagnosis of varicocele, and little difficulty is encountered in identifying large or moderate-sized varicoceles. The distended veins may appear as a vermiform bluish discoloration beneath the scrotal skin. Dubin and Amelar described the classical "bag of worms" appearance on palpation. Varicoceles can be described according to their size, as large or grade 3 when visible through the scrotal skin; moderate or grade 2 when easily palpable without a Valsalva maneuver, and small or grade 1 when palpable only with the

Valsalva maneuver. The physical examination has to be performed with the patient in both supine and upright positions. A warm and comfortable environment is also important.

***Adjunctive Diagnostic Tests:*** **Doppler.** A pencil-probe Doppler (9 MHz) can be used to aid in the diagnosis of the varicocele. With the patient in the upright position and performing the Valsalva maneuver, a venous "rush" is heard, and the clinical impression of a varicocele may be confirmed. Nevertheless, the significance of a positive test result in subfertile, oligospermic patients with a subclinical varicocele screened by Doppler is uncertain; this test should only be used as an adjuvant technique to confirm physical findings consistent with a varicocele or to examine the contralateral cord to determine if a hard-to-palpate varicocele exists.

**Ultrasonography.** The usefulness of ultrasonography in the evaluation of the subfertile male has been clinically demonstrated, especially in patients with a small scrotum or thickened scrotal skin that interferes with the diagnosis. For patients with recurrent or persistent varicoceles, a color-flow Doppler may be helpful. There is no consensus on the scientific merits of correcting a subclinical varicocele, nor is there agreement on whether one should extensively investigate suspected lesions in patients who have one.

**Venography**. Retrograde spermatic venography is the most sensitive technique for diagnosis of varicoceles; reflux is seen in almost 100% of patients with a varicocele. However, its specificity and significance in reference to the "subclinical" varicocele are unclear. Due to the invasive nature of venography, it should not be used routinely to diagnose varicoceles. The most appropriate indication for venography is the persistent varicocele in the postsurgical patient.

**Additional Techniques**. Scrotal thermography and scintigraphy with technetium-99m may be useful in supporting the diagnosis of clinically apparent varicoceles; however, these techniques should not be used to search for a subclinical varicocele.

### (b) Varicocele - General Concepts

Varicoceles are found in approximately 15% of the general population, including adolescents and adults,[31] in 35% of men with primary infertility (reported range: 19%-41%),[32] and in 80% of men with secondary infertility. Improvements in seminal variables, testicular size, and testicular histology have been observed after varicocelectomy.[33-35] Varicocele repair has been reported to improve spermatogenesis in 50%-80% of patients, and 30%-40% will initiate a pregnancy after the procedure.[36]

Clinically, varicoceles occur more commonly as isolated left-sided lesions (60%-90%). Although the incidence of bilateral varicoceles historically has been reported to be approximately 10%, more recent studies have shown bilaterality in 30%-50% of cases.[35,36] The isolated right-sided varicocele is uncommon and causes concern regarding the possibility of an underlying retroperitoneal abnormality, such as renal tumor with venous invasion.

The left internal spermatic vein drains perpendicularly into the left renal vein, taking a course that is approximately 8-10 cm longer than that of the right internal spermatic vein, which enters the inferior vena cava at an oblique angle. This unique venous insertion on the left can result in increased hydrostatic pressure, overcoming valvular mechanisms and resulting in venous backflow, venous dilatation, and ultimate varicocele formation.

Increased hydrostatic pressure within the left internal spermatic vein may also result from compression of the left renal vein between the superior mesenteric artery and the aorta, the so-called "nutcracker" effect.

Absence or incompetence of valves appears to play a role in the etiology of the varicocele.[37] Most varicoceles are thought to be due to abnormalities of the internal spermatic venous system. Nonetheless, dilatation of the external spermatic (cremasteric) system may be clinically significant, and it has been suggested that any vein greater than 4 mm in diameter should be ligated.[38]

### (c) Pathophysiology

There are several hypotheses to explain the mechanisms by which a unilateral varicocele may exert a bilaterally deleterious effect on spermatogenesis and fertility. These include renal and adrenal reflux, hypoxia, hormonal dysfunction, and hyperthermia.

***Renal and Adrenal Reflux:*** The reflux of metabolic products or by-products of the adrenal glands and kidneys has been hypothesized to have a toxic effect on testicular function. The pathophysiology of the varicocele remains to be clearly defined. The role of renal metabolites has not been adequately investigated.

***Hypoxia:*** It has been hypothesized that varicoceles cause hypoxia because of the stagnation of less oxygenated blood pooling around the testes. Hypoxia, indeed, may have a detrimental effect on spermatogenesis, but there is no evidence that varicoceles induce hypoxia.

***Hormonal Dysfunction:*** Decreased levels of plasma testosterone were found in patients with varicocele; this hypoandrogenic state may play a part in the effects of varicocele on spermatogenesis.[39] Other authors have shown that testosterone levels in venous or internal spermatic vein blood of patients with varicocele do not differ from those of normal men.

Also, a subtle alteration in the hypothalamic-pituitary-gonadal (HPG) axis can be found in some varicocele patients.[40] Whether this is the mechanism of the effect of the varicocele or the result of the primary pathophysiologic effect of the varicocele is unclear.

***Hyperthermia:*** Elevation of scrotal temperature is the factor most widely believed to explain the observed effects of the varicocele on spermatogenesis. In normal patients, intrascrotal temperatures are 0.6°-0.8°C lower than the intrascrotal temperatures of patients with varicoceles.[41] In patients with preoperative sperm concentrations of less than 50 million/mL and postoperative counts of greater than 50 million/mL, a decrease in scrotal temperatures of 0.5°C was found after varicocelectomy.[42]

### (d) Indications for Repair

***Infertility:*** Impaired fertility is the most common indication for varicocele repair. However, one must be certain that there are not other causes for the patient's infertility as well. Moreover, the mere presence of a varicocele in a subfertile male is not alone an indication for varicocele repair. The varicocele repair can improve parameters measured by the semen analysis and morphology measured by strict criteria.[43]

***Symptoms:*** Testicular or scrotal pain is not commonly experienced by patients with varicoceles; if pain is persistent, after other causes of pain are ruled out, a varicocele repair may be beneficial. Peterson et al. showed an 86% incidence of complete resolution of pain after varicocele ligation.[44] However, conservative measures, such as scrotal support and analgesics, should be offered first.

***Pediatric and Adolescent Varicocele:*** There are several reports in the literature indicating that the childhood varicocele may become apparent peripubertally and that early corrective therapy could prevent future damage to the individual's fertility status; however, this remains a controversial subject. As we noted previously,[45] the presence of a varicocele is associated with loss of testicular mass that appears to be progressive with age. After a pediatric or adolescent varicocele is corrected, a significant increase in testicular volume can be observed- a phenomenon called "catch-up" growth of the affected testis.[46]

Many authors have adopted the position that a pediatric varicocele should be left untreated unless there is significant testicular asymmetry or impaired testicular growth (>20% volume disparity).

***Secondary Infertility:*** The role of varicoceles in infertility in males with secondary infertility is generally agreed upon. Witt and Lipshultz, in 1993, determined that the varicocele is a progressive lesion that can result in loss of previously established fertility.[47]

### (e) Surgical Treatment

Surgical varicocele repair is the mainstay of varicocele therapy. The goal of intervention is the complete disruption of the internal spermatic venous drainage of the testicle without damaging the internal spermatic artery, vas deferens with its blood supply, and spermatic cord lymphatics.

Several surgical approaches have been used. These include the scrotal approach, the inguinal approach (modified Ivanissevich), the retroperitoneal approach (modified Palomo), and the laparoscopic approach.

The scrotal approach is rarely used. Because of the complexity of the anatomy of the scrotal pampiniform plexus it is possible to damage all three major sources of blood to the testis and epididymis: the spermatic, the deferential, and the cremasteric arteries.

The retroperitoneal approach involves an incision at the level of the internal inguinal ring, splitting of the external and internal oblique muscles, and exposure of the internal spermatic artery and vein retroperitoneally near the ureter. This technique minimizes the potential for recurrence of the varicocele, but does not allow identification and ligation of the external cremasteric vessels, which can be a cause of recurrent and persistent varicocele.[48,49]

The popularity of laparoscopy in general has led in recent years to its use for the ligation of the internal spermatic veins. As a retroperitoneal approach, its advantages and disadvantages are similar to those of the open retroperitoneal approach.

***Microscopic Varicocele Repair - Inguinal Approach:*** The inguinal approach exposes the internal spermatic vessels within the inguinal canal. As noted by Ivanissevich, this approach has several advantages over the retroperitoneal approach.[50] First, the urologic surgeon is more familiar with the anatomy within the inguinal canal. As the spermatic vein reaches the internal inguinal ring, it will coalesce to form several larger vessels. This approach will also allow for identification of large external cremasteric vessels that may contribute to the varicocele.

Traditional approaches to inguinal varicocelectomy involve a 5-10 cm incision over the inguinal canal, opening of the external oblique aponeurosis, and delivery of the spermatic cord.

Once the cord is exposed, the use of the operating microscope facilitates the dissection and reduces the likelihood of complications. The spermatic fascia is incised and teased apart to allow identification and isolation of the dilated veins. The testicular artery or arteries can be easily identified and avoided, and lymphatics can be easily visualized and preserved. The bleeding is minimized with more precise dissection under magnification and use of a microsurgical bipolar cautery.

Often, there is a typical pattern of the veins and artery with one or two large veins lying laterally and two smaller veins on either side of the artery more medially (Fig. 3). Use of the micro Doppler probe facilitates identification and confirmation of sparing of the testicular artery or arteries (Fig. 4). The Doppler tip is only 1-2 mm in diameter. Splaying the cord out over one's finger makes it possible for the vas deferens to be placed laterally and out of the area of dissection. Combining tactile with visual assessment helps to determine the extent of the varicocele.

### (f) Nonsurgical Treatment

Percutaneous Venous Occlusion: Techniques used to achieve transvenous occlusion have included use of detachable balloons, coils, and sclerotherapy. Proponents of the percutaneous technique support its use as a timely and cost-effective outpatient procedure. The overall success rate of this procedure is 69%.[32] We use embolization for the treatment of surgical failures or in patients with previous inguinal surgery who do not want another operation.

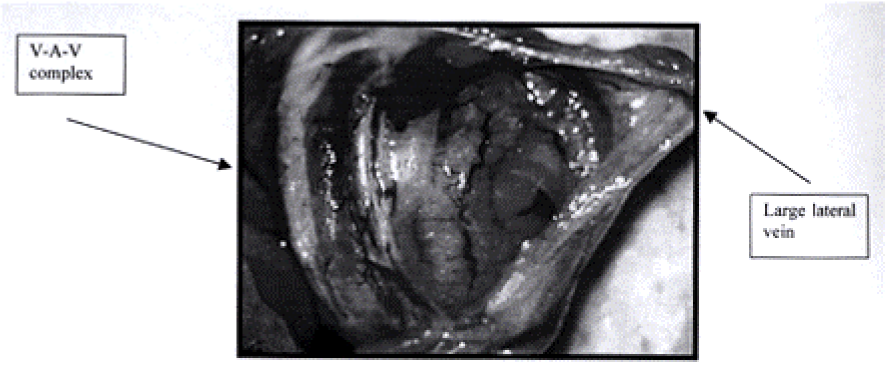

**FIGURE 3**. Varicocele vascular pattern.

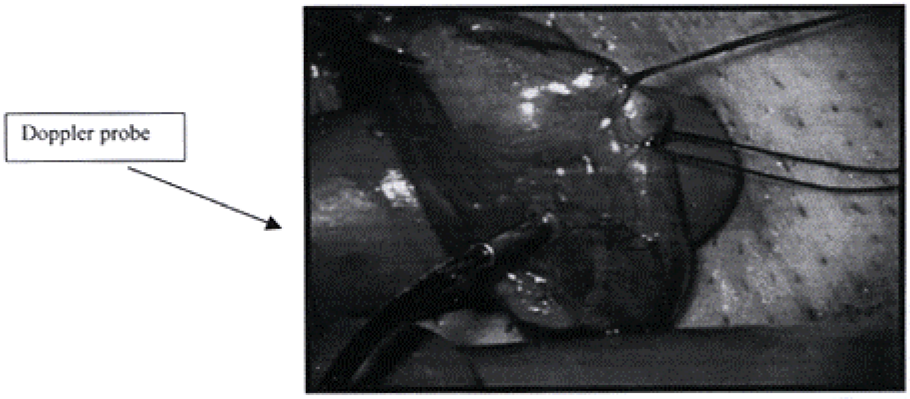

**FIGURE 4**. Micro Doppler probe used intraoperatively.

### *(g) Semen Improvement and Pregnancy Rates*

An accurate comparison of outcomes using different treatment modalities is difficult because of innumerable, inconsistently controlled variables. Although there are some series that show no significant effect or an adverse effect of varicocele repair, these series are relatively small and may be biased as well as statistically misinterpreted (Tables 6&7).[51-53] A review of 15 papers[32] showed an overall rate of improvement in semen quality of 66% (range 51%-78%) and an overall reported pregnancy rate of 43% (range 24%-53%).

A randomized, prospective study of patients with varicoceles, comparing those treated surgically with those treated by observation alone has been reported by Madgar et al.[54] The results clearly demonstrated that varicocelectomy improves sperm quality and fertility rates. In this study the average pregnancy occurred 6-9 months following surgery.

An extensive review of the literature concluded that varicocelectomy has a beneficial effect on sperm density.[55] This effect seems more pronounced when initial semen densities are greater than 10 million/mL. It has also been reported that motility and morphology may improve after varicocelectomy concomitant with an associated rise in density, although isolated improvements in either of these parameters have also been cited. Similarly, several authors have shown the improvement in sperm morphology measured by strict criteria.[43,56]

**TABLE 6**
**Studies Demonstrating Improvement in Semen Quality and Pregnancy Rate After Varicocelectomy**

| SELECTED STUDIES | YEAR | NO. OF PATIENTS | IMPROVED SEMEN QUALITY | PREGNANCY RATE (%) |
|---|---|---|---|---|
| Scott & Young [82] | 1962 | 166 | 70 | 31 |
| Charny & Baum[83] | 1968 | 104 | 61 | 24 |
| MacLeod[41] | 1965 | 108 | 74 | 41 |
| Brown[84] | 1976 | 251 | 58 | 41 |
| Dubin & Amelar[36] | 1977 | 986 | 70 | 53 |
| Newton *et al*.[85] | 1980 | 149 | 66 | 34 |
| Marks *et al*. [57] | 1986 | 130 | 51 | 39 |
| Goldstein *et al*.[86] | 1992 | 357 | - | 43 |
| Ross & Ruppman[87] | 1993 | 488 | 77 | - |
| Madgar *et al*.[54] | 1995 | 45 | - | 71 |

Modified from Nagler, H.M., Luntz, R.K., and Martinis, F.G.: Varicocele. In: *Infertility in the Male* , 3rd Edition. Edited by L.I. Lipshultz and S.S. Howards. St. Louis: Mosby-Year Book, 1997, p.337.

**TABLE 7**
**Studies Demonstrating No Beneficial Effect of Varicocelectomy on Pregnancy Rates**

| Selected Studies | Year | Pregnancy Rate (%) After Varicocelectomy | Pregnancy Rate (%) With No Surgery |
|---|---|---|---|
| Nilsson *et al*.[52] | 1979 | 8 | 18 |
| Baker *et al*.[51] | 1985 | 47 | 45 |
| Vermeulen *et al*.[53] | 1986 | 24 | 56 |

Modified from Nagler, H.M., Luntz, R.K., and Martinis, F.G.: Varicocele. In: *Infertility in the Male* , 3rd Edition. Edited by L.I. Lipshultz and S.S. Howards. St. Louis: Mosby-Year Book, 1997, p.337.

In an attempt to identify prognostic factors that could predict postoperative pregnancies, Marks, MacMahon, and Lipshultz described a cohort of 130 patients and identified four useful prognostic variables.[57] The absence of testicular atrophy was found to indicate a good prognosis; 56% of patients with normal testicular size established pregnancies, compared with a 33% pregnancy rate in patients with testicular atrophy. An initial sperm count greater than 50 million/ejaculate was also associated with a higher pregnancy rate. Patients with a normal motility established a pregnancy in 60% of cases, whereas only 30% of patients with abnormal motility were successful. An elevation of FSH was a poor prognostic indicator. Only 25 % of patients whose FSH was above 300 ng/mL (normal <300 ng/mL) achieved pregnancies, whereas 46% of patients with normal FSH concentration had successful outcomes.

### *(h) Complications*

The most common complication of varicocele repair is recurrence or persistence of the varicocele. After surgical treatment, the recurrence rate has been reported to be between 0% and 20%.[32,58,59]

Operative complications of incisional surgery include incidental testicular artery ligation with subinguinal approaches (<2% in microdissection cases) and occasional damage to the ilioinguinal nerve within the inguinal canal. Also, hematomas and scrotal edema may be seen in up to 13% of cases involving a microsurgical inguinal repair, while a higher rate of hydrocele formation is seen with the retroperitoneal approach.[60] The most important complications related to the type of surgery are shown in Table 8.

**TABLE 8**
**Techniques of Varicocelectomy**

| TECHNIQUE | ARTERY PRESERVED | HYDROCELE (%) | FAILURE (%) | POTENTIAL FOR SERIOUS MORBIDITY |
|---|---|---|---|---|
| **Microscopic inguinal approach** | yes | 0 | 0.5 | yes |
| **Laparoscopic** | yes | 10 | 15 | yes |
| **Retroperitoneal** | no | 7 | 15-25 | no |
| **Conventional inguinal** | no | 3-39 | 5-15 | no |

Modified from Nagler, H.M., Luntz, R.K., and Martinis, F.G.: Varicocele. In: *Infertility in the Male* , 3rd Edition. Edited by L.I. Lipshultz and S.S. Howards. St. Louis: Mosby-Year Book, 1997, p.337.

### *(i) Varicocele Repair in Azoospermic Patients*

A new and very exciting concept is that of performing varicocele repair for azoospermic patients. A primary benefit of varicocele repair in azoospermic men with spermatogenic failure is the possibility of promoting production of motile sperm in the ejaculate. When a choice is possible, using motile sperm from a fresh ejaculate is preferable to using testicular sperm extraction in preparation for ICSI and IVF.[61] Freshly ejaculated sperm are associated with ICSI success rates superior to those achieved with sperm retrieved via testicular sperm extraction, and, additionally, an invasive and potentially damaging procedure is avoided.[62]

Recently several independent investigators have reported that varicocelectomy can result in significant improvement in sperm density and total number of motile sperm in 40%-50% of azoospermic men with spermatogenic failure.[63-65]

### *(j) Treatment of Pituitary Tumors*

Hyperprolactinemia has been described in patients with idiopathic oligospermia as well as in patients with prolactin-secreting pituitary tumors. The presence of a pituitary tumor in a subfertile male may be suggested by serum hormonal abnormalities (i.e., elevated prolactin, low testosterone without increase in LH) or clinical findings (i.e., impaired libido, visual field changes). In such cases, appropriate radiographic evaluation of the pituitary fossa is indicated and transsphenoidal surgical ablation performed when necessary. The use of bromocriptine in these patients is discussed elsewhere.

### Procedures to Improve Sperm Delivery

### (a) Vasovasostomy

Occlusion of the excretory ducts of the testis is an important entity because of its potential reversibility. The incidence of ductal obstruction among infertile men has been reported as 7.4%. The causes of ductal obstruction include congenital absence of the ductal system, ductal stricture following infection, vasectomy, and functional obstruction.

The ductal system may be congenitally absent. Usually, there is an associated absence of the seminal vesicles, ampulla, vas deferens, and a major portion of the epididymis. Unilateral renal agenesis has also been noted in some affected men. CF, a rare disorder of infancy, is almost always associated in males with congenital hypoplasia or absence of these excretory ducts. Because of the absence of seminal vesicles, these patients have, in addition to azoospermia, a low ejaculate volume, and semen that does not coagulate at the time of ejaculation. Recent studies have now shown that patients with vasal agenesis may also be carriers of one of several CF genes.[66,67]

By contrast, stricture of the excretory ducts acquired following infection of these structures may be remediable. Prior to the antibiotic era, gonorrheal urethritis frequently progressed to an obstructive epididymitis. At present, gonorrhea is an unusual cause of male infertility. Vasoepididymal anastomoses may successfully bypass the obstruction and restore normal fertility potential to men with this lesion. Currently, vasectomy is the leading cause of infertility secondary to ductal obstruction.

***Technique:*** There are many recognized methods of performing a vasovasostomy; however, the experience of the surgeon is obviously the most important component. Our bias is that a more precise operation can be performed with some type of magnification, but a strict two-layer anastomosis may not be necessary, although this is our procedure of choice. We present a technique of vasal reanastomosis employing the operating microscope and a two-layer anastomosis.

With the patient preferably under general anesthesia, a vertical scrotal incision is made, and the testicle, epididymis, and proximal vas deferens are bluntly dissected free from surrounding tissue without opening the tunica vaginalis. The site of vasectomy is palpated and a towel clamp passed under it, taking care to avoid damage to the vas deferens. The vas proximal to the site of vasectomy is then sharply freed from surrounding tissue. The proximal (testicular) end of the vas is then perpendicularly cut with an empty scalpel handle and #11 blade scalpel or with a nerve cutter. Fluid from the testicular end of the transected vasal lumen is aspirated with a syringe and Angiocath. The fluid is then microscopically examined for the presence of sperm as well as for its opacity and thickness. If no sperm, no fluid, or thick, paste-like fluid is encountered, then more proximal exploration of the vas and/or epididymis is indicated. If whole sperm, sperm with short tails, or clear, thin fluid is encountered, reanastomosis of the testicular vas deferens at this level is performed. Sharp dissection of the vas distal to the previous dissection site is then done. Above the vasectomy site, the vas is isolated with a towel clamp, sharply dissected free from surrounding tissue, and transected in a perpendicular fashion, as previously described. The distal lumen is very gently dilated with jeweler's forceps. The patency of the distal segment may be confirmed quickly and easily by irrigation with saline via a syringe and Angiocath. Bleeding is carefully controlled with bipolar cautery.[68]

The tissue adjacent to the proximal (testicular) vas is next approximated to the tissue adjacent to the distal (abdominal) vas with a 4-0 PDS suture. The suture is positioned in such a way that a tension-free, end-to-end anastomosis can be performed.

Initially, a 9-0 nylon suture is placed at the 6 o'clock position of the testicular vas incorporating the serosa and superficial muscularis mucosa. The suture is then placed in the 6 o'clock position of the abdominal end of the vas incorporating the same tissue planes. This suture aligns the testicular and abdominal ends of the vas as well as takes tension off the mucosal anastomosis to be performed. Next, utilizing double-armed, 10-0 nylon suture, a stitch is placed in the 6 o'clock position of the testicular vas mucosa in an inside-to-outside fashion. The opposite needle of the double-armed, 10-0 suture is placed through the mucosa of the abdominal vas at the 6 o'clock position, again proceeding from inside to outside. This stitch is then tied down. Two additional 10-0 sutures are placed in a similar fashion in the 4 o'clock and 8 o'clock positions and tied down.

The mucosal layer is completed with 3ñ5 additional 10-0 sutures, as described for the initial mucosal suture. It is easiest to leave the anterior mucosal sutures to be tied at one time, after they have all been placed. After tying all of the mucosal sutures, the anastomosis is examined for leakage. If a mucosal leak is identified, it is repaired with 10-0 nylon in an inside-to-outside fashion. The mucosal anastomosis is reinforced with a layer of seromuscular 9-0 sutures, as described above, that do not enter the lumen. The second layer begins anteriorly and usually requires 10-12 sutures circumferentially. Bipolar cautery is utilized to obtain hemostasis. The testis and vasal structures are then replaced within the scrotum, and the dartos muscle and skin are closed with 4-0 chromic gut suture in separate layers. The same procedure is repeated on the contralateral side. Drains are used if bleeding has been excessive, and they are removed the following day.

In a review of 1,247 men undergoing vasectomy reversal, Belker et al. related the time of obstruction to success of the surgery (Table 9).[69]

**TABLE 9**
**Obstuctive Interval vs. Postoperative Results After Vasectomy Reversal**

| YEARS OBSTRUCTED | NO. WITH SPERM (%) | NO. PREGNANT (%) |
|---|---|---|
| **Fewer than 3** | 86/89(97) | 56/74(76) |
| **3-8** | 525/600(88) | 253/478(53) |
| **9-14** | 32/45(71) | 11/37(30) |

Modified from Thomas, A.J., Jr., and Howards, S.S.: Microsurgical treatment of male infertility. In: *Infertility in the Male*, 3rd Edition. Edited by L.I. Lipshultz and S.S. Howards. St. Louis, Mosby-Year Book, 1997, p.377.

### *(b) Epididymovasostomy*

In most cases, vasal exploration is initially performed. If no sperm, no vasal fluid, or thick, opaque fluid is found in the vas, proximal obstruction is likely. The tunica vaginalis is then opened and the epididymis is examined. Induration or fullness of the epididymis grossly suggests obstruction. The overlying tunica vaginalis is dissected off the underlying dilated epididymal tubules with sharp dissection. Care must be taken to avoid inadvertently entering the tubules during this dissection. Microscissors are then used to create a circular epididymotomy in the side of an engorged epididymal tubule. Visualization of the epididymotomy is aided by the use of indigo carmine. The epididymal fluid is then aspirated and examined microscopically. If whole sperm are found, the anastomosis is performed at this level. Motile sperm are cryopreserved. If sperm are absent, more proximal epididymal exploration is performed (Table 10). When whole sperm are found, an end-to-side epididymovasostomy is done incorporating the existing epididymotomy and the vasal lumen distal to the site of vasal obstruction (Fig. 5-8).[70,71] Double-armed, 10-0 nylon is utilized to place sutures in the 3 o'clock, 6 o'clock, 9 o'clock, and 12 o'clock positions in an inside-to-outside fashion (Fig. 9).[70,71]

**TABLE 10**
**Epididymovasostomy Success Rates, Scott Department of Urology, Baylor College of Medicine**

| INTRAOPERATIVE FINDINGS | | | RESULTS | |
|---|---|---|---|---|
| **Sperm Present** | **Fluid Quality** | **Procedure** | **Patency Rate** | **Pregnancy Rate** |
| Yes | Good | Vasovasostomy | 95% | 75% |
| No | Good | Vasovasostomy | 60% | 50% |
| No | Poor* | Epididymovasostomy | 50% | 45% |

*Absent or pasty

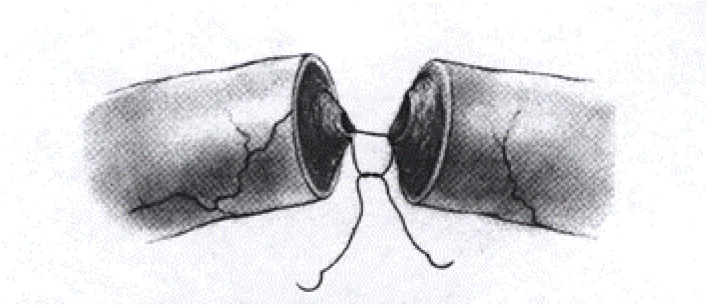

**FIGURE 5.** The first mucosal 10-0 suture of a formal two-layer anastomosis. (Fig. 5-8 Modified from Thomas, A.J., and Howards, S.S.: Microsurgical treatment of male infertility. In: *Infertility in the Male*, 3rd edition. Edited by L.I. Lipshultz and S.S. Howards. St.Louis, Mosby-Year Book, 1997, p.376.)

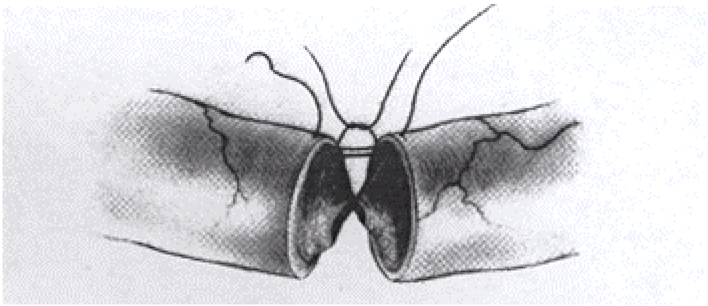

**FIGURE 6**. Two 8-0 sutures initiating an outer layer of a formal two-layer anastomosis.

The distal vas is prepared in a fashion similar to that for a vasovasostomy procedure. The vas distal to the site of obstruction is sharply dissected free from surrounding tissue. The vas is then transected perpendicularly with an empty scalpel handle and #11 blade scalpel or with a nerve cutter. The abdominal end of the transected vas is then flushed with saline to check for distal patency. If no evidence of distal obstruction exists, the tissue adjacent to the abdominal end of the vas is approximated to the tissue adjacent to the epididymis with a 4-0 PDS suture. Further approximation of the vasal lumen to the epididymotomy is performed with 7-0 and 9-0 nonabsorbable sutures incorporating the seromuscular layers of the vas and the tunica vaginalis of the epididymis.

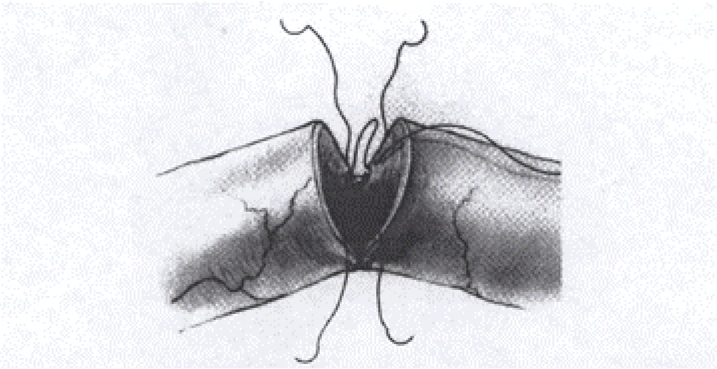

**FIGURE 7**. Posterior row has been completed. The vas-holding clamp has been turned, and three 10-0 luminal sutures are placed. One can use sutures with needles on either end, as shown in the illustration, or cut the sutures in half. It is easier to leave the last two or three sutures unties until they are all placed.

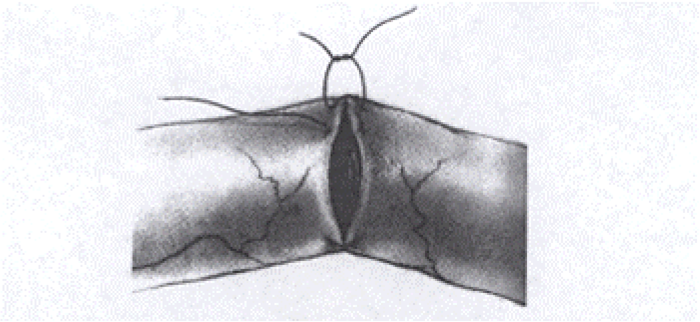

**FIGURE 8**. The outer layer is completed with 8-0 sutures.

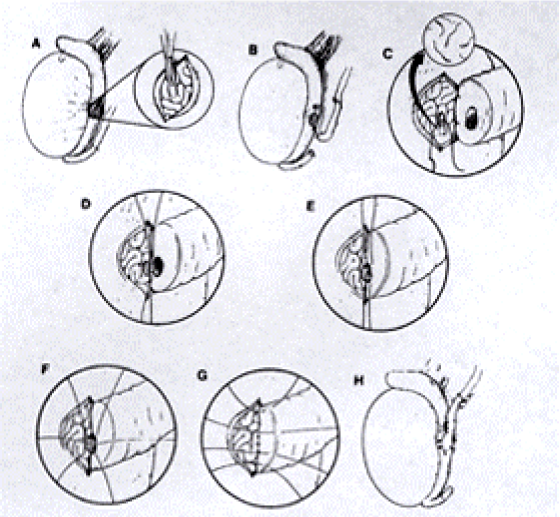

**FIGURE 9**. Method of "end-to-side" single tubule vasoepididymostomy. (Modified from Thomas, A.J., Jr., and Howards, S.S.: Microsurgical treatment of male infertility. In: *Infertility in the Male*, 3rd Edition. Edited by L.I. Lipshultz and S.S. Howards. St. Louis, Mosby-Year Book, 1997, p.381)

With the use of the previously placed 10-0 nylon sutures in the epididymis, the anastomosis is created by placing each stitch in a symmetric location on the vasal lumen, that being the 3 o'clock, 6 o'clock, 9 o'clock, and 12 o'clock positions, respectively. Each suture is placed in an inside-to-outside fashion utilizing the opposite swedged needle from that used on the epididymal lumen. The anastomosis is then checked for leakage; if a leaking point is identified, another 10-0 nylon suture is used to close it. The anterior seromuscular vas is then approximated to the anterior tunica vaginalis of the epididymis. After completion of the anastomosis, the testis is returned to the scrotum, which is closed in separate layers with chromic gut suture. Again, drains are freely used when indicated. This procedure usually takes about 3-4 hours and can be done on an outpatient basis. The patient is asked to refrain from strenuous activity and sexual intercourse for 3 weeks. Semen analyses are performed 4 weeks after surgery and every 3 months for the first year.

A new technique, published by Berger in 1998, describes a triangulation end-to-side vasoepididymostomy with good results, being an alternative method of vasoepididymostomy.[72]

### (c) Incision of Ejaculatory Ducts

In evaluating patients with azoospermia or severe oligospermia, obstruction at the level of the ejaculatory ducts must be ruled out. Other findings consistent with this diagnosis are low semen volumes and palpably present vas deferens. Diagnosis of ejaculatory duct obstruction is suggested by TRUS;[73,74] dilation of the ampulla of the vas deferens to the level of the ejaculatory duct is consistent with this diagnosis. In patients who have low-volume azoospermia, dilation of the distal vas to the level of the ejaculatory duct on TRUS, and no evidence of sperm in the postejaculate urine, transurethral incision of the ejaculatory ducts is indicated. This diagnosis may also be made at the time of scrotal exploration and antegrade vasography.

### (d) Microscopic Epididymal Sperm Aspiration (MESA)

MESA, popularized in 1988, is a technique for the procurement of sperm from the epididymis of men in whom transport of sperm from the testicle to the ejaculate is not possible because the drainage (ductal) system is absent or is not subject to reconstruction. This problem most commonly occurs in men with vasal agenesis, a condition in which the vas deferens or drainage system of the testicle fails to develop prior to birth. Sixty to eighty percent of these patients will have mutations of the CF gene. Their wives will also require CF testing. The obstructive problem, however, also may be acquired, as after a failed epididymovasal anastomosis; here again, MESA may be indicated. While the male is being evaluated as a candidate for MESA, his wife is being screened in an IVF program where micromanipulation is also offered. MESA and IVF or micromanipulation complement each other and must be part of a coordinated program.

Epididymal aspiration may be performed on the day of the wife's ovulation, or preferably ahead of time, and the aspirated sperm cryopreserved. A scrotal incision is made and the epididymis identified microscopically and opened. Sperm are directly aspirated from a single, isolated epididymal tubule until 10-20 million are obtained.[24] The entire procedure is performed through an operating microscope. When the aspiration is completed, the epididymal tubule is closed with a microsurgical technique, and the scrotum is closed in a routine surgical fashion. The recovery period is approximately 24 hours.

The success of pregnancy from this procedure is reported to be 25%-40%. It is a complex process, requiring significant manipulation of the human gametes (eggs and sperm) but one that offers a previously sterile couple the chance of establishing a pregnancy using their own genetic material.

***Percutaneous Epididymal Sperm Aspiration (PESA):*** This has been reported. While this procedure may be appropriate in some circumstances (such as when a patient has an obstruction and only desires one child), published fertilization rates have not been as high as those from MESA. In addition, the blind nature of the procedure raises the risk of damage to the epididymis or testis.

### *(e) Testicular Sperm Extraction (TESE)*

In approximately 7% of men undergoing MESA, spermatozoa retrieval is not possible. In these patients in whom the epididymal sperm reservoir is inaccessible, recent work has shown that whole, motile sperm can be obtained from the testicle itself. Critical for the success of TESE is determination that the testis is making mature sperm. This can be confirmed by diagnostic testicular biopsy, which is sent for touch preparation (cytology), image analysis, and standard histology. If mature sperm with complete tails are present on these examinations, TESE is feasible. Because of the low number of sperm obtained with TESE, these sperm need to be combined with the wife's eggs using ICSI.

***Patients who require TESE can be divided into two categories:***

1. The first set of patients is those men whose epididymis is not accessible because of previous epididymal procedures or congenital efferent ductal obstruction in the testis.
2. Patients with testicular biopsies demonstrating predominantly Sertoli-cell-only, maturation arrest, or severe hypospermatogenesis represent another group of patients in whom testicular sperm retrieval has been used, if areas of normal sperm production can be demonstrated in a testicular biopsy, as described by Su et al., in 1999.[75]

In order to obtain testicular tissue from which to perform TESE, a standard testicular biopsy is performed. In patients with nonobstructive azoospermia, several biopsy specimens may need to be obtained from each testis in order to find areas of active spermatogenesis. This can usually be performed through one small incision on each side of the scrotum. This testicular tissue is then sent to the IVF laboratory, where it is microdissected and processed in order to obtain sperm. Although in the past, fresh TESE was the only option, some authors have described the use of frozen TESE, with good results.[76]

In patients with obstructive azoospermia, TESE is close to 100% successful at retrieving sperm if diagnostic biopsies have previously demonstrated sperm in the testis. In the nonobstructed testicle, recent studies have shown that 86% and 91% of patients will have some sperm obtained by TESE. Pregnancy rates ranging between 28% and 50% per cycle of ICSI have been reported recently for patients with nonobstructive azoospermia. Sperm can also be aspirated, i.e., testicular sperm aspiration (TESA), and this technique can be used as an effective alternative to MESA.[77]

### NON-SURGICAL TREATMENT OF THE INFERTILE MALE

### Specific Therapy[78]

### *(a) Hypogonadotropic Hypogonadism*

Hypogonadotropic hypogonadism is the cause of infertility in a small percentage of patients and can be either congenital or acquired. Congenital hypogonadotropic hypogonadism (Kallmannís syndrome) results from the abnormal production or secretion of gonadotropin-releasing hormone (GnRH) by the hypothalamus and may be associated with other congenital anomalies such as anosmia, deafness, cleft palate, and renal anomalies.[79] Causes of acquired hypogonadotropic hypogonadism include pituitary tumors, isolated gonadotropin deficiency, panhypopituitarism, pituitary trauma, and anabolic steroid use.

The initial evaluation of a patient with hypogonadotropic hypogonadism should include a head CT or MRI to rule out the possibility of a pituitary tumor that may require surgical extirpation. In addition, a serum prolactin level should be obtained and hyperprolactinemia, if present and not requiring surgical treatment of a tumor, medically treated prior to initiation of gonadotropin replacement therapy.

In patients with gonadotropin deficiency, normal spermatogenesis can frequently be restored by treatment with exogenous gonadotropins or GnRH. Human chorionic gonadotropin (hCG), which possesses LH-like activity, and human menopausal gonadotropin (hMG), which contains both FSH and LH, have been the replacement agents used most frequently in the treatment of these patients. Treatment usually is begun with hCG (1,500-3,000 I.U.) administered subcutaneously three times per week. After 8-12 weeks of hCG therapy, hMG therapy is added at a dose of 37.5-150 I.U. administered subcutaneously 2-4 times per week. Serum testosterone levels and semen analyses are followed serially during the course of treatment.

Treatment of hypogonadotropic hypogonadism with pulsatile GnRH analogs has been reported with similar success. Whitcomb and Crowley reported restoration of fertility in 8 of 9 patients with idiopathic hypogonadotropic hypogonadism using pulsatile GnRH therapy.[80] The GnRH is given either intravenously or subcutaneously in a pulsatile fashion with a portable infusion pump or intranasally. Patients who fail to respond to hCG/hMG therapy may respond to GnRH therapy. In addition, patients with hypogonadotropic hypogonadism who fail to respond to conventional gonadotropin therapy or GnRH agonists may respond to a combination of growth hormone and gonadotropins.

### *(b) Hyperprolactinemia*

Excessive production of prolactin by the posterior pituitary has been implicated as a cause of both infertility and erectile dysfunction. In one series of male infertility patients, 4% were noted to have elevated serum prolactin levels. Prolactin has a negative influence on the hypothalamic secretion of gonadotropin releasing hormone and is thought to have an inhibitory effect on LH binding to Leydig cells in the testis. Patients with hyperprolactinemia have decreased or normal serum gonadotropin levels and decreased testosterone levels.

The causes of hyperprolactinemia include pituitary tumors (macroadenoma or microadenoma), hypothyroidism, liver disease, and drugs, such as phenothiazine, tricyclic antidepressants, and some antihypertensives. All patients with hyperprolactinemia should undergo diagnostic imaging of the pituitary gland with either CT or MRI to rule out the presence of a pituitary macroadenoma. The treatment of hyperprolactinemia is based on the etiology. In patients with hypothyroidism, thyroid replacement by itself will usually reduce serum prolactin levels to the normal range. When medications are the cause, they should be discontinued when possible. Patients with functional macroadenomas of the pituitary generally require surgical therapy.

Medical therapy for patients with idiopathic hyperprolactinemia and microadenomas of the pituitary utilizes the dopaminergic agonist bromocriptine. Doses may vary from 2.5-7.5 mg per day given 2-4 times per day to avoid gastrointestinal side effects and can be increased with time. In most cases, the serum prolactin levels will return to normal.

### *(c) Isolated Testosterone Deficiency*

In rare instances, hypogonadism may result from isolated testosterone deficiency (primary hypogonadism) secondary to Leydig cell failure. Restoration of the testosterone level to normal is possible with intramuscular depot preparations of testosterone (testosterone enanthate or testosterone cypionate) and results in improvement of libido and sexual function. Unfortunately, the probability of restoring normal spermatogenesis in these patients is low but they may respond to hCG (1,500 I.U. t.i.w.).

### (d) Congenital Adrenal Hyperplasia (CAH)

CAH has been reported to be a cause of male factor infertility. In CAH patients, deficiencies in adrenal hydroxylase result in decreased cortisol secretion. The resulting increase in pituitary ACTH secretion leads to increased adrenal androgen production and, thus, to feedback that inhibits secretion of gonadotropins from the pituitary and suppresses spermatogenesis. The diagnosis can be made by demonstrating increased urinary 17-ketosteroids or dehydroepiandrosterone (DHEA). The condition is treated with glucocorticoid replacement.

Although the diagnosis usually is made in childhood, several cases of infertility secondary to CAH have been reported in adult men. These patients generally have responded to glucocorticoid replacement with increased sperm production. It should be noted that many patients with untreated CAH have been shown to have normal seminal parameters. Although a rare entity, CAH should be considered in the differential diagnosis of male subfertility.

### (e) Immunologic Infertility

An immunologic basis for some cases of infertility was demonstrated as early as the 1950s. Recently, a great deal of interest has been focused on research into possible immunologic causes of infertility and the treatment of these disorders. While the use of oral Prednisone to decrease significant ASA titers has been advocated, it is rarely successful. ICSI is the treatment of choice for serious immunological male factor problems.

### (f) Genital Tract Infection

At one time, genital tract infection was considered to be a significant cause of male subfertility. However, genital tract infection associated with abnormal semen quality as an isolated abnormality was noted in only 1.6% of 2,871 subfertile couples in a multicenter study reported in 1986. Postinfectious causes of male infertility have been well-documented.

Testicular atrophy may occur following severe epididymo-orchitis with *Enterobacteriaceae*, *Chlamydia trachomatis*, or *Neisseria gonorrhoeae*. Epididymal duct obstruction has been observed occasionally as a result of epididymitis, and prostatitis seldom has been associated with ejaculatory duct obstruction.

Pyospermia (>1 million WBC/mL) is not common in subfertile patients. Association with infections is rare. Inflammation is the most likely causative factor, and these patients should be treated with nonsteroidal anti-inflammatory drugs. It is always a good idea to culture the semen first, but usually only common organisms are identified. Elevated white blood cells are significant because of their ability to generate ROS, which can harm the sperm's ability to fertilize.

Evaluation of the male patient with symptoms of genital tract infection and treatment of culture-proven genital tract infection are prudent actions. The empiric use of antibiotic agents in cases of idiopathic subfertility, however, is of questionable benefit, and in light of potential harmful effects of many antibiotic agents on spermatogenesis, should be discouraged.

### (g) Disorders of Ejaculation

To understand disorders of ejaculation, it is necessary to understand the physiology of ejaculation. A detailed description of the neurophysiology of ejaculation is beyond the scope of this presentation, and the reader is referred elsewhere for this information. The ejaculatory event is comprised of seminal emission and ejaculation. Seminal emission refers to the deposition of semen into the posterior urethra by contraction of the vasa deferentia and seminal vesicles. These events are mediated by the sympathetic nervous system via T10-L3 level preganglionic fibers. Ejaculation is the forceful expulsion of semen from the posterior urethra out the urethral meatus in an antegrade fashion. This event is secondary to the rhythmic contraction of periurethral and pelvic floor smooth muscles, mediated by parasympathetic (S2,3,4) outflow and somatic efferents, and occurs in conjunction with closure of the bladder neck, which is sympathetically stimulated. Disorders of ejaculation include: (1) retrograde ejaculation, (2) anejaculation, (3) retarded ejaculation, and (4) premature ejaculation. Only the first two entities will be discussed.

***Retrograde Ejaculation:*** Retrograde ejaculation can be defined as the abnormal backward flow of semen into the bladder with ejaculation. The etiology may be either anatomic, neurogenic, pharmacologic, or idiopathic. Anatomic causes include prostatectomy (open or transurethral) or bladder neck surgeries (Y-V plasty, transurethral incision of the bladder neck). Neurogenic causes include spinal cord injury, retroperitoneal surgery, and diabetes mellitus. Pharmacologic agents implicated in causing retrograde ejaculation include neuroleptics, tricyclic antidepressants, alpha-blockers used in the treatment of prostatism, and certain antihypertensives.

The diagnosis of retrograde ejaculation is made by examination of a postejaculate urine (PEU). The patient is instructed to void immediately following ejaculation. A sample of the specimen is evaluated microscopically for sperm density and motility. Alternatively, the specimen may be centrifuged and the pellet resuspended in a medium, such as human tubular fluid, and then analyzed for sperm density and motility.

In the treatment of retrograde ejaculation, an initial trial of medical therapy is warranted. The agents commonly utilized are alpha-adrenergic agonists such as ephedrine, pseudoephedrine, Imipramine, and phenylpropanolamine. Dosage schedules for these agents are shown in Table 11.

**TABLE 11**
**Pharmacologic Therapy for Retrograde Ejaculation**

| Medication | Dosage |
|---|---|
| Phenylpropanolamine | 75 mg b.i.d. x 10 days |
| Pseudoephedrine hydrochloride | 60 mg q.i.d. x 10 days |
| Ephedrine sulfate | 50 mg q.i.d. x 10 days |
| Imipramine hydrochloride | 25 mg b.i.d. x 10 days |

When medical therapy fails to restore antegrade ejaculation, recovery of sperm from the bladder after ejaculation in conjunction with intrauterine insemination (IUI) has been used. The patient is treated with oral bicarbonate (650 mg q.i.d. for 2 days) prior to sperm retrieval in order to alkalinize the urine. After ejaculation, the specimen is voided and then processed for insemination. An alternative but more invasive method involves catheterizing the patient, rinsing the bladder with 30 cc of a buffered medium, which is discarded, and then instilling an additional 30 cc of medium that is left indwelling. Immediately after ejaculation, the patient voids or is catheterized to retrieve the specimen. The procedure is timed to coincide with the wife's ovulation.

***Anejaculation:*** Anejaculation is a relatively uncommon disorder that can occur as a result of spinal cord injury, retroperitoneal lymph node dissection, or other retroperitoneal surgery, diabetes mellitus, transverse myelitis, or multiple sclerosis. In addition, the etiology may be psychogenic or idiopathic.

If medical therapy fails, anejaculation is now being treated with rectal probe electroejaculation (RPE) and penile vibratory stimulation. Initially used in veterinary medicine and animal husbandry, RPE has been used successfully in the treatment of human anejaculation for over 25 years. Initial results in terms of the ability to obtain motile sperm were variable, but with refinements in the equipment and techniques, sperm can now be obtained in approximately 90% of anejaculatory patients. These patients have excellent sperm densities averaging 180-300 million/cc. Despite successes in the induction of ejaculation, sperm motility has continued to be the limiting fertility factor in these patients. Mean sperm motilities of 20% have been reported in several series. This poor motility appears to be secondary to intrinsic factors such as elevated scrotal temperature and recurrent genitourinary tract infection rather than to the electroejaculation procedure. The use of assisted reproductive techniques, especially ICSI, has become increasingly important in this patient population.

Because these patients often have supranormal sperm densities with low motility, semen processing with Percoll gradients may be beneficial in removing non-motile sperm, white blood cells, and debris before insemination. Unfortunately, the poor motility of most of these specimens limits the potential use of sperm cryopreservation.

### Empiric Therapy

Assessing the use of empiric therapy for male infertility patients is difficult. Idiopathic oligospermia occurs in an extremely heterogeneous group of patients. Moreover, there has been no uniformity in the patient selection criteria for the reported studies. Many studies define oligospermia as a sperm density less than 20 million/cc, but others use a number above or below this. Some studies include patients with abnormal serum gonadotropin levels while others exclude them. Medications vary in dosage and timing from study to study. And, finally, in a situation unique in medicine, treatment outcome depends on two patients instead of one. Frequently, evaluation of the female factor is cursory and results are simply reported as "normal." However, in some studies the evaluation is extensive. The duration of infertility is also an important consideration, but mean infertility rates in reported studies range from 2-6 years.

**The importance of including a placebo control arm in any study involving a treatment for infertility cannot be overemphasized**. A large number of recent infertility studies that have utilized a control arm have demonstrated significant increases in seminal parameters during the course of the study in both the treatment and placebo groups. This effect has been postulated to be secondary to the patientís presentation for infertility evaluation at a time when sperm counts were at a nadir, with improvement to baseline occurring subsequently. In addition, the background or spontaneous conception rates have been shown to be as high as 25% in a number of placebo-controlled studies. This obviously casts doubt on earlier uncontrolled fertility studies that demonstrated a significant effect for a variety of medical treatments.

**Drugs that fall under the category of "empiric therapy" include:**

1. Clomiphene citrate
2. Tamoxifen
3. Androgens
4. Testosterone rebound
5. Aromatase inhibitors
6. Gonadotropins
7. GnRH
8. Kallikreins
9. Prostaglandin synthetase inhibitors
10. Bromocriptine
11. Pentoxifylline
12. Antioxidants
13. Carnitine

Only clomiphene citrate and antioxidants will be discussed in this review.

### (a) Anti-Estrogens

***Clomiphene Citrate:*** Clomiphene citrate is a synthetic, nonsteroidal anti-estrogen that is structurally related to diethylstilbestrol (DES). It competitively binds to the estrogen receptors in the hypothalamus and pituitary, thereby blocking feedback inhibition on these organs and effectively increasing the secretion of GnRH, FSH, and LH.

Clomiphene citrate is prescribed in doses of 12.5-50 mg per day and may be given continuously or on a 25-day cycle with a 5-day "rest period" each month. Monitoring of serum gonadotropins and testosterone is essential to make certain that the testosterone level remains within the normal limits, since higher levels may have a negative influence on spermatogenesis. In addition, because a small percentage of patients experience a decline in sperm density and motility while taking this medication, frequent semen analyses are mandatory.

The side effects of oral clomiphene therapy include gynecomastia, nausea, mild weight gain, dizziness, visual complaints, and allergic dermatitis. These side effects have been noted to occur in fewer than 5% of patients in controlled studies and are mild when they do occur.

Numerous investigators have studied the effect of clomiphene on male infertility patients since the initial studies by Mellinger and Thompson in 1966.[81] While many of these studies demonstrated improvement in seminal parameters and/or pregnancy rates with clomiphene in oral doses from 25-100 mg/day, few of these studies were placebo-controlled. In the past several years, well-designed, prospective, randomized, placebo-controlled studies with clomiphene have been performed (Table 12).

Controversy still exists with regard to the efficacy of clomiphene citrate for the treatment of male factor infertility. It is possible that a select group of patients responds to clomiphene therapy. It is also conceivable that by some as yet undetermined mechanism clomiphene improves pregnancy rates without improving bulk seminal parameters or sperm fertilizing capacity. Although it is difficult to predict which patients will respond to clomiphene, patients not likely to benefit from this therapy include those with elevated baseline serum FSH levels, severe oligospermia, asthenospermia, teratozoospermia or azoospermia, and those with extremely abnormal testis biopsy results.

### (b) Antioxidants

Another area of interest in the study of idiopathic male subfertility is the potential effect of ROS on sperm function and fertility. ROS include hydroxyl radical (OH), superoxide anion (O2) and hydrogen peroxide (H2O2). These compounds appear to be generated by both seminal leukocytes and sperm cells. ROS can induce damage by peroxidation of sperm lipid membranes and creation of toxic fatty acid peroxides. Sperm axonemes also appear to be the targets of ROS-induced sperm damage. Human sperm are particularly sensitive to damage by these reactive oxygen compounds because of the high content of polyunsaturated fatty acids contained in the sperm membrane. Human semen possesses several scavenging systems to counteract the potentially harmful effects of ROS including the enzymes superoxide dismutase (SOD), glutathione peroxidase, and catalase.

Recently, several studies have demonstrated an increase in ROS in the semen of men with idiopathic subfertility. The ability of sperm to exhibit sperm-oocyte fusion has been noted to be inversely related to seminal levels of ROS. In light of these studies, several investigators have attempted to introduce compounds into the semen that detoxify or scavenge these ROS.

Vitamin E was found to be an effective antioxidant that significantly improved the capacity for sperm-oocyte fusion *in vitro*. Typically used oral doses of vitamin E (400-1,200 I.U. per day) recently have been shown to be insufficient to increase the seminal plasma levels to those used in the in vitro studies. Pentoxifylline treatment of sperm in vitro has been noted to decrease sperm production of superoxide anion by 50%. Platelet-activating factor (PAF), lyso-PAF, and lysophosphatidyl choline have been shown to increase sperm motility and forward progression in vitro and have been postulated to act as ROS scavengers.

**TABLE 12**
**Controlled Studies of Clomiphene Citrate as Empiric Therapy**

| Author (Year) | Clomiphene Dosage Control Drug | Duration (Months) | Subjects | Pregnancy Rates (%) |
|---|---|---|---|---|
| Foss (1973)[88] | 100 mg/day<br>Placebo | 3<br>3 | 108<br>101 | 17** |
| Paulson (1979)[89] | 25 mg/day<br>Cortisone, 10 mg/day | 6<br>6 | 20<br>20 | 35<br>10 |
| Ronnberg (1980)[90] | 50 mg/day<br>Placebo | 3<br>3 | 27<br>29 | 10<br>3 |
| Abel (1982)[91] | 50 mg/day<br>Vitamin C, 200 mg/day | 9<br>9 | 98<br>89 | 17<br>13 |
| Wang (1983)[92] | 25 mg/day<br>50 mg/day<br>Placebo | 6-9<br>6-9<br>6-9 | 11<br>18<br>7 | 36<br>22<br>0 |
| Micic (1985)[93] | 50 mg/day<br>No treatment | 6-9<br>6-9 | 56<br>45 | 13<br>0 |
| Sokol (1988)[94] | 25 mg/day<br>Placebo | 12<br>12 | 23<br>23 | 9<br>44 |
| Check (1989)[95] | 25 mg/day<br>Placebo | 8<br>8 | 50<br>50 | 58<br>16 |
| WHO (1992)[96] | 25 mg/day<br>Placebo | 6<br>6 | 94<br>96 | 8<br>12 |

* these studies did not report improvement in bulk seminal parameters in a consistant manner.
**Overall pregnancy rate for clomiphene and placebo groups combined

A recent placebo-controlled trial of the antioxidant glutathione in the treatment of oligospermic subfertile males with varicoceles or pyospermia revealed improvements in sperm motility and morphology. Further research is needed to determine if agents such as these will be clinically useful either for the in vitro treatment of sperm or as orally administered systemic agents for treatment of a subgroup of men with increased levels of seminal fluid ROS or decreased ROS scavenging.

### REFERENCES

1. Goldenberg, R.L., and White, R.: The effect of vaginal lubricants on sperm motility in vitro. Fertil. Steril., 26:872, 1975.
2. Johnson, A., Bassham, B., Lipshultz, L.I., et al.: A quality control system for the optimized sperm penetration assay. Fertil. Steril., 64(4), 1995.
3. Puri, P., Barton, D., OíDonnell, B.: Prepubertal testicular torsion: Subsequent fertility. J. Pediatr. Surg., 20:598, 1985.
4. Werner, C.A.: Mumps orchitis and testicular atrophy. Ann. Intern. Med., 32:1066, 1950.
5. Strohmer, H., et al.: Agricultural work and male infertility. Am. J. Ind. Med., 24:587, 1993.

6. Lantz, G.D., et al.: Recovery of severe oligospermia after exposure to dibromochloropropane (DMCP). Fertil. Steril., 35:46, 1981.
7. Benoff, S., et al.: The effect of calcium ion channel blockers on sperm fertilization potential, Fertil. Steril., 62:606, 1994.
8. Schormeyer, T., Knuth, U.A., and Belken, L.: Reversible azoospermia induced by anabolic steroid 19-nortestosterone. Lancet, 1:417, 1984.
9. Rock, J., and Robinson, D.: Effects of induced intrascrotal hyperthermia on testicular function in man. Am. J. Obstet. Gynecol., 793, 1965.
10. Foster, R.S., et al.: The fertility of patients with clinical stage I testis cancer managed by nerve-sparing retroperitoneal lymph node dissection. J. Urol., 152:1139, 1984.
11. Lubs, H.A., Jr.: Testicular size in Klinefelterís syndrome in men over fifty. N. Engl. J. Med., 267:326, 1962.
12. Saypol, D.C., Lipshultz, L.I., and Howards, S.S.: Varicocele. In: Infertility in the Male, First Edition. Edited by L.I. Lipshultz and S.S. Howards. New York: Churchill Livingstone, 1983.
13. Anderson, D.J.: Semen white blood cell assay In: Infertility in the Male, 3rd Edition. Edited by L.I. Lipshultz and S.S. Howards. St. Louis: MosbyñYear Book, 1997, p. 509.
14. Kruger, T.F., et al.: Predictive value of abnormal sperm morphology in in vitro fertilization. Fertil. Steril., 49:112, 1988.
15. Jeyendran, R.S., Van Der Ven, H.H., and Zaneveld, L.J.D.: The hypo-osmotic swelling test: An update. Arch. Androl., 29:105, 1992.
16. Niederberger, C.S., Lamb, D.J., Glinz, M., Lipshultz, L.I., and Scully, N.F.: Tests of sperm function for evaluation of the male: Penetrak and Tru-Trax. Fertil. Steril., 60:319ñ323, 1993.
17. Smith, R.G., et al.: Functional tests of spermatozoa. Sperm penetration assay. Urol. Clin. North Am., 14:451, 1987.
18. Rao, M., and Rao, D.: Cytogenetic studies in primary infertility. Fertil. Steril., 28:209, 1977.
19. Griffin, J.E., and Wilson, J.D.: Disorders of sexual differentiation. In Walsh, P.C., et al. (eds.): Campbellís Urology, Volume 2. Edited by P.C. Walsh, et al. Philadelphia: W.B. Saunders, 1992.
20. Charny, C.W.: Testicular biopsy: Its value in male sterility. J.A.M.A., 115:1429, 1940.
21. Hotchkiss, R.S.: Testicular biopsy in the diagnosis and treatment of sterility in the male. Bull. N.Y. Acad. Med., 18:600, 1942.
22. Rajfer, J., and Binder, S.: Use of Biopty gun for transcutaneous testicular biopsies. J. Urol., 142:1021, 1989.
23. Jequier, A.M., Crich, J.P., and Holmes, S.C.: Incomplete obstruction of the male genital tract: A cause of oligozoospermia. Br. J. Urol., 55:545, 1983.
24. Schlegel, P.N., et al.: Epididymal micropuncture with in vitro fertilization (IVF) and oocyte micromanipulation for treatment of unreconstructable obstructive azoospermia. Fertil. Steril., 61:895, 1994.
25. Skakkebaek, N.E.: Possible carcinoma-in-situ of the testis. Lancet, 2:516, 1972.
26. Netto, N.R., Jr.: Needle method of testicular biopsy. Int. Surg., 59:172, 1974.
27. Cohen, M.S., et al.: Testicular needle biopsy in diagnosis of infertility. Urology, 24:439, 1984.
28. Abyholm, T., and Clausen, O.P.F.: Clinical evaluation of DNA flow cytometry of fine needle aspirates from testes of infertile men. Int. J. Androl., 4:505, 1981.
29. Skakkebaek, N.E., et al.: Quantification of human seminiferous epithelium. III. Histological studies in 44 infertile men and controls with normal chromosome complements. Acta Path. Microbiol. Scand., 81:97, 1973.
30. Coburn, M., Wheeler, T.M., and Lipshultz, L.I.: Cytological examination of testicular specimens. Abstract of the American Fertility Society Annual Meeting, 1986.
31. Belloli, G., DíAgostino, S., Pesce, C., and Fantuz, E.: Varicocele in childhood and adolescence and other testicular anomalies: An epidemiological study. Pediatr. Med. Chir., 15:159, 1993.
32. Pryor, J.L., and Howards, S.S.: Varicocele. Urol. Clin. North Am., 14:499, 1987.
33. Cockett, A.T.K., Urry, R.L., and Dougherty, K.A.: The varicocele and semen characteristics. J. Urol., 121:435, 1979.
34. Kass, E.J., and Belman, A.B.: Reversal of testicular growth by varicocele ligation. J. Urol., 137:475, 1987.
35. Saypol, D.C., Lipshultz, L.I., and Howards, S.S.: Varicocele. In: Infertility in the Male, 1st Edition. Edited by L.I. Lipshultz and S.S. Howards. New York: Churchill Livingstone, 1983.
36. Dubin, L., and Amelar, R.D.: Varicocelectomy: 986 cases in a 12 year study. Urology, 10:446, 1977.
37. Ahlberg, N.E., Bartley, O., and Chidekel, N.: Right and left gonadal veins: An anatomic and statistical study. Acta Radiol. Diagn., 4:593, 1966.
38. Chehval, M.J., and Purcell, M.H.: Varicocelectomy: Incidence of external spermatic vein involvement in the clinical varicocele. Fertil. Steril., 39:573, 1992.
39. Comhaire, F., and Vermeulen, A.: Plasma testosterone in patients with varicocele and sexual inadequacy. J. Clin. Endocrinol. Metab., 40:824, 1982.
40. Okuyama, A., et al.: Pituitary-gonadal function in schoolboys with varicocele and indications for varicocelectomy. Eur. Urol., 7:92, 1981.
41. MacLeod, J.: Seminal cytology in the presence of varicoceles. Fertil. Steril., 16:735, 1965.
42. Agger, P.: Scrotal and testicular temperature: Its relation to sperm count before and after operation for varicocele. Fertil. Steril., 22:286, 1971.

43. Schatte, E.C., Hirshberg, S.J., Fallick, M.L., Lipshultz, L.I., and Kim, E.D.: Varicocelectomy improves sperm strict morphology and motility. J. Urol., 160(4):1338ñ1340, 1998.
44. Peterson, A.C., Lance, R.S., and Ruiz, H.E.: Outcomes of varicocele ligation done for pain. J. Urol., 159(5):1565ñ1567, 1998.
45. Lipshultz, L.I., Corriere, J.N., Jr.: Progressive testicular atrophy in the varicocele patient. J. Urol., 117:175, 1977.
46. Lemach, G.E., Uzzo, R.G., Schlegel, P.N., and Goldstein, M.: Microsurgical repair of the adolescent varicocele. J. Urol., 160(1):179ñ181, 1998.
47. Witt, M.A., and Lipshultz, L.I.: Varicocele: A progressive or static lesion? J. Urol., 42:541, 1993.
48. Coolsaet, B.L.: The varicocele syndrome: Venography determining the optimal level for surgical management. J. Urol., 124:833, 1980.
49. Murray, R.R., Jr., et al.: Comparison of recurrent varicocele anatomy following surgery and percutaneous balloon occlusion. J. Urol., 135:286, 1986.
50. Ivanissevich, O.: Left varicocele due to reflux; experience with 4.470 cases in forty-two years. J Int. Coll. Surg., 34:742, 1960.
51. Baker, H.W.G., Burger, H.G., and Dekretser, D.M.: Testicular vein ligation and fertility in men with varicoceles. Br. Med. J., 291:1678, 1985.
52. Nilsson, S., Edvinsson, A., and Nilsson, B.: Improvement of semen and pregnancy rate after ligation and division of the internal spermatic vein: Fact or fiction? Br. J. Urol., 51:591, 1979.
53. Vermeulen, A., Vanderweghe, M.N., and Deslypere, J.P.: Prognosis of subfertility in men with corrected or uncorrected varicocele. J. Androl., 7:147, 1986.
54. Madgar, I., et al.: Controlled trial of high spermatic vein ligation for varicocele in infertile men. Fertil. Steril., 63:120, 1995.
55. Schlesinger, M.S., and Nagler, H.M.: Treatment outcome after varicocelectomy. Urol. Clin. North Am., 21:517, 1994.
56. Vazquez-Levin, M.H., Friedmann, P., Goldberg, S.I., Medley, N.E., and Nagler, H.M.: Response of routine semen analysis and critical assessment of sperm morphology by Kruger classification to therapeutic varicocelectomy. J. Urol., 158(5):1804ñ1807, 1997.
57. Marks, J.L., MacMahon, R., and Lipshultz, L.I.: Predictive parameters of successful varicocele repair. J. Urol., 136:609, 1986.
58. Hommonai, Z.T., Fainman, N., Engelhard, Y., Rudberg, Z., David, M.P., and Paz, G.: Varicocelectomy and male fertility: Comparison of semen quality and recurrence of varicocele following varicocelectomy by two techniques. Int. J. Androl., 3:447ñ456, 1980.
59. Szabo, R., and Kessler, R.: Hydrocele following internal spermatic vein ligation: A retrospective study and review of the literature. J. Urol., 132:924ñ925, 1984.
60. Ito, H., Kotake, T., Hamano, M., et al.: Results obtained from microsurgical therapy of varicocele. Urol. Int., 51:227ñ227, 1993.
61. Palermo, G., Joris, H., Devroey, P., and Van Steirteghem, A.C.: Pregnancies after intracytoplasmic injection of single spermatozoon into an oocyte. Lancet, 340:17, 1992.
62. Aboulghar, M.A., Mansour, R.T., Serour, G.I., Fahmy, I., Kamal, A., Tawab, N.A., and Amin, Y.M.: Fertilization and pregnancy rates after intracytoplasmic sperm injection using ejaculate semen and surgically retrieved sperm. Fertil. Steril., 681:108, 1997.
63. Esteves, S.C., and Glina, S.: Recovery of spermatogenesis after microsurgical varicocele repair in azoospermic men is related to testicular histology. Abstract #1199. Presented at AUA 94th Annual Meeting, Dallas, Texas, May 1ñ6, 1999.
64. Kim, E.D., Leibman, B.B., Grinblat, D.M., and Lipshultz, L.I.: Varicocele repair improves semen parameters in azoospermic men with spermatogenic failure. J. Urol., 162(3):737ñ740, 1999.
65. Matthews, G.J., Matthews, E.D., and Goldstein, M.: Induction of spermatogenesis and achievement of pregnancy after microsurgical varicocelectomy in men with azoospermia and severe oligoasthenospermia. Fertil. Steril., 70(1):71ñ75, 1998.
66. Anquiano, A., et al.: Congenital bilateral absence of the vas deferens. A primarily genital form of cystic fibrosis. J. Androl., 15:1, 1994.
67. Oates, R.D., and Amos, J.A.: The genetic basis of congenital bilateral absence of the vas deferens and cystic fibrosis. J. Androl., 15:1, 1994.
68. Thomas, A.J., Jr., and Howards, S.S.: Microsurgical treatment of male infertility. In: Infertility in the Male, 3rd Edition. Edited by L.I. Lipshultz and S.S. Howards. St. Louis: MosbyñYear Book, 1997, pp. 371ñ384.
69. Belker, A.M., et al.: Results of 1,469 microsurgical vasectomy reversals by the Vasovasostomy Study Group. J. Urol., 145:505, 1991.
70. Thomas, A.J., Jr.: Vasoepididymostomy. Urol. Clin. North Am., 14:527, 1987.
71. Thomas, A.J., Jr.: Microsurgical end-to-side vasoepididymostomy: An analysis of 153 procedures. Paper presented at the 88th Annual Meeting of the American Urological Association, San Antonio, Texas, May 1993.
72. Berger, R.E.: Triangulation end-to-side vasoepididymostomy. J. Urol., 153(6):1951ñ1953, 1998.
73. Belker, A.M., and Steinbock, G.S.: Transrectal prostate ultrasonography as a diagnostic and therapeutic aid for ejaculatory duct obstruction. J. Urol., 144:356, 1990.

74. Carter, S.S.C., Shinohara, K., and Lipshultz, L.I.: Transrectal ultrasonography in disorders of the seminal vesicles and ejaculatory ducts. Urol. Clin. North Am., 16:773, 1989.
75. Sul, M., Palermo, G.D., Goldstein, M., Veeck, L.L., Rosenwaks, Z., and Schlegel, P.N.: Testicular sperm extraction with ICSI for nonobstructive azoospermia. Testicular histology can predict success of sperm retrieval. J. Urol., 161(1):112ñ116, 1999.
76. Oates, R.D., Mulhall, J., Colleen, B., Cunningham, D., and Carson, R.: Fertilization and pregnancy using intentionally cryopreserved testicular tissue as the sperm source for ICSI in 10 men with non-obstructive azoospermia. Hum. Reprod., 12(4):734ñ739, 1997.
77. Lisek, E.W., and Levine, L.A.: Percutaneous technique for extraction of sperm from the epididymis and testicle. Tech. Urol., 3(2):81ñ86, 1997.
78. Gilbaugh, J.G., III, and Lipshultz, L.I.: Nonsurgical treatment of male infertility. An update. Urol. Clin. North Am., 21(3):531, 1994.
79. Honig, S.C., and Oates, R.D.: Infertility. In: Clinical Urology. Edited by R.J. Krane, M.B. Siroky, and J.M. Fitzpatrick. Philadelphia: J.B. Lippincott, 1994.
80. Whitcomb, R.W., and Crowley, W.F.: Male hypogonadotropic hypogonadism. Endocrinol. Metab. Clin. North Am., 22:125, 1993.
81. Mellinger, R.C., and Thompson, R.J.: The effect of clomiphene citrate in male infertility. Fertil. Steril., 17:94, 1966.
82. Scott, L.S., and Young, D.: Varicocele: A study of its effect on human spermatogenesis and of the results produced by spermatic vein ligation. Fertil. Steril., 13:335, 1962.
83. Charny, C.W., and Baum, S.: Varicocele and infertility. J.A.M.A., 204: 1165, 1968.
84. Brown, J.S.: Varicocelectomy in the subfertile male: A ten year experience with 295 cases. Fertil. Steril., 27:1046, 1976.
85. Newton, R., Schinfeld, J.S., and Schiff, I.: The effect of varicocelectomy on sperm count, motility, and conception rate. Fertil. Steril., 34:250, 1980.
86. Goldstein, M., Gilbert, B.R., Dicker, A.P., Dwosh, J., and Gnecco, C.: Microsurgical inguinal varicocelectomy with delivery of the testis: An artery and lymphatic sparing technique. J. Urol., 148: 1808ñ1811, 1992.
87. Ross, L.S., and Ruppman, N.: Varicocele vein ligation in 565 patients under local anesthesia: A long term review of technique, results and complications in light of proposed management by laparoscopy. J. Urol., 149:1361, 1993.
88. Foss, G.L., Tindall, V.R., and Birkett, J.P.: The treatment of subfertile men with clomiphene citrate. J. Reprod. Fertil., 32:167, 1973.
89. Paulson, D.F., et al.: Clomiphene citrate: Pharmacologic treatment of hypofertile men. Urology, 9:419, 1977.
90. Rànnberg, L.: The effect of clomiphene citrate on different sperm parameters and serum hormone levels in preselected infertile men: A controlled double-blind cross-over study. Int. J. Androl., 3:479, 1980.
91. Abel, B.J., et al.: Randomized trial of clomiphene citrate treatment and vitamin C for male infertility. Br. J. Urol., 54:780, 1982.
92. Wang, C., et al.: Serum bioactive follicle-stimulating hormone in men with idiopathic azoospermia and oligospermia. J. Clin. Endocrinol. Metab., 65:629, 1987.
93. Micic, S., and Dotlic R.: Evaluation of sperm parameters in clinical trial with clomiphene citrate of oligospermic men. J. Urol., 133:221, 1985.
94. Sokol, R.Z., et al.: A controlled comparison of the efficacy of clomiphene citrate in male infertility. Fertil. Steril., 49:865, 1988.
95. Check, J.H., et al.: Empirical therapy of the male with clomiphene in couples with unexplained infertility. Int. J. Fertil., 34:120, 1989.
96. World Health Organization: A double-blind trial of clomiphene citrate for the treatment of idiopathic male infertility. Int. J. Androl., 15:299, 1992.

**This article should be referenced as follows:**

Moreira, S.G. and Lipshultz, L.I. (2004) Management of male infertility. *TheScientificWorldJOURNAL* **4 (S1)**, 214–248.

**Handling Editor:**

Anthony Atala, Principle Editor for *Urology* — a domain of *TheScientificWorldJOURNAL*.
